# Application of plant viruses in biotechnology with relation to artificial intelligence, machine learning, and nanotechnology

**DOI:** 10.1038/s44298-026-00193-6

**Published:** 2026-07-10

**Authors:** Khushboo Jain, Anchal Saini, Avinash Marwal, Sunny Dhir, S. M. Paul Khurana

**Affiliations:** 1 Department of Biotechnology, Faculty of Science, MVSC, Janardhan Rai Nagar Rajasthan Vidhyapeeth (Deemed to be University), Udaipur, India; 2 Advanced Center for Climate Resilience & Crop Protectio, Maharishi Markandeshwar (Deemed to be University), Ambala, India; 3 Nanoparticle Synthesis and Bioinformatics Laboratory, Department of Biotechnology, Vigyan Bhawan, Block - B, New Campus, Mohanlal Sukhadia University, Udaipur, India; 4 Amity University, Gurgaon, India

**Keywords:** Biological techniques, Biotechnology, Computational biology and bioinformatics, Microbiology, Plant sciences

## Abstract

Plant viruses, once viewed as harmful agricultural pathogens, are now powerful tools in biotechnology. Their nanoscale structure, self-assembly, and biocompatibility enable applications in agriculture, medicine, and environmental sustainability. They serve in gene delivery, genome editing, diagnostics, and nanomaterials for vaccines and drug delivery. Integration with AI, ML, and bioinformatics enhances virus discovery and prediction. Despite challenges, plant viruses are emerging as versatile, sustainable resources for global biotechnological innovations.

## Introduction and overview of plant viruses

Plant viruses have been known in the past for their destructive impact on global agriculture. They have been primarily responsible for a significant decline in crop yields, food insecurity, and an economic burden. It is estimated that plant viruses are responsible for 10–15% of the total agricultural yield loss worldwide, with extremely serious consequences for the production of rice, wheat, maize, sugarcane, and tomatoes^1^. Their ability to mutate quickly, exchange genetic material, and infect new hosts has turned them into a continuous source of danger for both poor and rich farmers. On the other hand, the story about plant viruses has changed a lot during the past 10 years. Consequently, they are shifting from being solely pathogens to being recognised as one of the most promising and naturally occurring nanomaterials for the development of modern biotech^2^. This change in the way we look at things is supported by the unique combination of features that are typical of plant viruses: nanoscale precision, self-assembly, structural uniformity, ease of production, genetic tractability, and environmental stability. These characteristics allow viral capsids or virus-like particles (VLPs) to be considered as the support for molecular display, the delivery vehicles which can be programmed, or the functional nanomaterials for sensing, catalysis and targeted therapy^3^. Moreover, the biocompatibility and the inability of plant viruses to replicate in mammalian systems, which is a remarkable safety feature, make them even more attractive as the agents of human therapy and diagnostics^4^.

The changes in the natural characteristics of the plants were not the only major changes. Simultaneously with these changes in the biological attributes, the quantum leaps of artificial intelligence (AI), machine learning (ML), and bioinformatics have taken plant virus studies to an unimaginable level. Research using AI-based image recognition systems has brought plant virus identification to the field level by common devices like smartphones and drones, besides the already-existing hyperspectral imaging technology^5^. ML algorithms can be used to predict a viral protein structure, locate interaction points between the host and virus, and build a model for possible future evolutions, thus guiding the right preventive measures against viruses in crops and the creation of new virus strains for this purpose^6^. The bioinformatics area has been changed dramatically by next-generation sequencing (NGS) and the establishment of genomic data processing pipelines. With such methods, plant virus genomes can be sequenced and annotated in no time, resulting in a great expansion of viral diversity previously unrecognised^7^. The use of computers and related technologies to study nature is very helpful in tracing the viral evolution and epidemiology, as well as in constructing synthetic viral vectors and changing the capsid for delivery. Concurrently, nanotechnology has opened the doors to change the plant virus structure to one that is usable in the real world. Virus-based nanoparticles (VNPs) and VLPs from such viruses as Tobacco mosaic virus (TMV), Potato virus X (PVX), and Cowpea mosaic virus (CPMV) have been restructured as drug delivery nanocarriers, imaging probes, and vaccines^8,9^. Furthermore, heating and chemical treatments may change the characteristics, the surface and the volume of the cargo of the virus, allowing the researchers to design custom nanoscale systems for medical and agricultural applications^3^. In agriculture, these nanocarriers are being studied for delivering the pesticides, the nematicides, and the growth regulators in a precise manner to reduce the environmental load and increase the efficiency of the products used^10^.

The merging of plant virology, AI/ML, bioinformatics, and nanotechnology has come far to mean the crossing of an entirely new research frontier. The field of plant virology, which had been mainly concerned with disease control, is now working alongside computational biology and nanoscale engineering, which, in turn, has made plant virology relevant to the areas of medicine, environmental sustainability, and industrial biotechnology. For instance, TMV-based nanoparticles are being designed for cancer immunotherapy^11^, whereas geminivirus-derived vectors are the ones that are facilitating accurate genome editing in crops^12^. On top of that, AI-enabled biosensors consisting of plant VNPs fused with predictive algorithms are being created for quick viral detection in fields of agriculture, which is indispensable equipment in stopping the outbreak of diseases in the face of climate change^13^. Despite such advancement, there are still some difficulties. Problems with mass production, getting the green light from regulatory bodies, ensuring the safety of the biosphere for a long period of time, and the lack of standardised computational pipelines are the major issues impeding the widespread use of plant virus-based technologies. Still, the expanding number of transversal research works signals a future that is not so far away from one in which plant viruses would not be considered simply as barriers to the productivity of agriculture, but as smart biological resources of the biotech industry, helping the world to solve the hunger, healthcare, and environmental sustainability problems.

### Definition and classification of plant viruses

Plant viruses are biological entities whose genomes can be composed of either RNA or DNA (the RNA or DNA can be single or double-stranded). The replication process and the spreading are done entirely with host cellular machinery. These genomes are usually very concise and encode only a limited number of proteins, but they can establish quite complex control of the host interactions, movement, and transmission. To date, more than a thousand species of viruses that infect plants in various groups have been identified and described. The International Committee on Taxonomy of Viruses (ICTV) categorises them primarily according to the type of the genome, the strandedness, the replication method, the morphology of the capsid, the range of hosts and the mode of vectors. The main families of plant viruses are *Potyviridae* (positive-sense RNA viruses), *Geminiviridae* (single-stranded DNA, circular), *Tombusviridae, Bromoviridae, Caulimoviridae*, etc. Transmission is generally by vector-mediated (insects, aphids, whiteflies, leafhoppers), nematodes, or by mechanical means. Classification is not merely descriptive: it also indicates which genetically modifiable viruses are those that have the desired capsid structures and are the safest in terms of host range and biosafety. TMV, PVX, and geminiviruses, for example, are frequently used as tools in vector-based expression systems or in VLP applications because of their well-known genomes, ease of manipulation, and relatively broad knowledge of host interactions^14–16^.

### Characteristics of plant viruses

Several biological and physicochemical properties make plant viruses particularly suitable for exploitation in biotechnology, especially when combined with computational and nanotechnological techniques:

- **Structural stability and self-assembly:** viruses that infect plants in various ways often build protective coats, or protein shells, which remain very stable under different environmental conditions. Without any outside help, they can make their structures that are identical in size and shape (for example, they can be rods, icosahedra, or filamentous forms). The properties of these capsids allow for the cells inside to be changed (by genetics or chemistry) and to have other molecules attached to the surface, whether it is the inside or the outside. As a matter of fact, virus-shaped rods such as TMV and flexible, filamentous particle PVX are the substances used in nanomaterials and the scaffolds of tissue engineering^17^^.^
- **Genetic simplicity with engineering potential:** plant viruses are easier to manipulate as their genomes are small and have few genes. Scientists are able to construct virus vectors to carry a foreign gene, gene editing tools, or reporter genes. VLPs may be non-infectious but still hold their particular structural or functional characteristics. For instance, Tobacco mild green mosaic virus TMGMV-derived nanocarriers were changed with polycations to provide more effective DNA delivery into plant cells (inactivated TMGMV-PAH), thus, transient gene expression without infectivity risk was obtained^9^.
- **Capability for systemic movement and cell-to-cell spread:** numerous plant viruses can transport themselves inside plants (through plasmodesmata, vascular tissue), which enables them to be delivered systemically with genetic or protein payloads. Such an ability for movement is taken advantage of in plant immunisation, molecular farming, and in the use of engineered vectors for metabolic change or trait modification^12^.
- **Biocompatibility and safety:** viruses of the plant type normally are unable to productively replicate in mammalian cells^4^. These viruses are non-pathogenic to mammals and thus can be used as safer carriers for health applications in humans, such as vaccine adjuvants, immunotherapy, and drug delivery. Studies have shown that plant virus nanoparticles (PVNPs) can be recognized by the mammalian immune system, which may be advantageous for vaccine applications but necessitates thorough safety profiling for other therapeutic uses^18^. Importantly, their production in plants offers a cost-effective and scalable manufacturing platform with reduced risk of mammalian pathogen contamination^19^. Besides, their manufacturing in plants might be a cheaper and more scalable process.
- **Adaptability with computational and nanotechnological integration:** computational modelling, data-driven design (using ML/AI), and high-throughput bioinformatics analysis are possible due to the modular nature of their structure and genome. The areas of application span from the creation of diagnostic tools (biosensors) to the development of predictive models for virus emergence and their interaction with the host, and the design of the capsid functionalization for targeted delivery. A case in point, AI/ML techniques were employed to comprehend the pathogenesis, forecast the origin of diseases, and facilitate the diagnostics of plant viruses^20,21^.
- **Nanoparticle properties and functionalization:** VLPs and viral nanoparticles (VNPs) that come from plant viruses have been made functionally decorated, engineered, or changed in their shape (for example, a thermal shape shifting from rod to sphere) for delivering agrochemicals, displaying vaccines, presenting antigens, or for an application of soil mobility. Such changes make it possible to adjust the surface properties, aspect ratios, charge, cargo loading, and so on, thus allowing customised performance^3,22^.

### Objective of the study

This article aims to describe the latest and most complete coverage of the application of plant viruses in biotechnological fields, especially those that intersect with AI, ML, bioinformatics, and nanotechnology. Most of these areas have been extensively developed independently; however, the combined consideration of virology, computational biology, and nanotech is necessary to fathom how these fields modally interact and co-accelerate innovation. In this paper, the authors intend to transform the image of plant viruses from a threat to agriculture into reliable carriers for different domains of biotechnology, mainly as promoters of the data science and nanoscale engineering era.

## Leveraging plant viruses for next-gen biotech innovations

Plant viruses, once thought of strictly as pathogens, have become a beneficial resource in biotechnology. Their intrinsic ability to enter plant cells, replicate, and move systemically provides a special advantage in delivering genetic materials, silencing particular genes, and making targeted changes to genomes. These strengths provide the potential to use plant viruses in gene delivery systems, viral transformation vectors, and genome editing in several plant species^12,23^. Different plant viruses have already been used in this capacity. RNA virus vectors such as TMV, PVX, and Barley stripe mosaic virus (BSMV) have already been modified and utilised as vectors capable of temporarily expressing foreign genes or silencing host genes via virus-induced gene silencing (VIGS)^24^. These systems are fast and do not require the production of transgenic plants, making them extremely valuable for generating data through high-throughput gene function studies. DNA viruses act as replicon-based vectors, and especially Geminiviruses like Bean yellow dwarf virus (BeYDV) and African cassava mosaic virus (ACMV), replicate their genomes to very high numbers in the host nucleus. These replicons can be used to deliver CRISPR/Cas9 components and be effective platforms for homology-directed repair (HDR)^25–27^). Viral vectors have advantages over other transformation methods. They necessarily require no regeneration or tissue culture; gene expression is transient, fast, and can be obtained in good quality for foresight in several different plant species^12^. These characteristics make plant viruses powerful tools for functional genomics, metabolic engineering, and high-throughput screening. TMV, PVX, BSMV, and engineered geminivirus genes growing as transgenes or RNA silencing in plants^28^. Each system has a suite of host plants for specific applications. In summary, substantial progress has been made in VIGS, and the use of plant viruses for delivering genome editing components (e.g., CRISPR/Cas systems)^29^. Furthermore, VIGS, transgenic expression, silencing through small RNAi, and also editing the host susceptibility gene, allow for proof of principle of such applications in virus-based biotechnology to generate crops resistant to viruses.

### Gene delivery systems

**Fig. 1:**
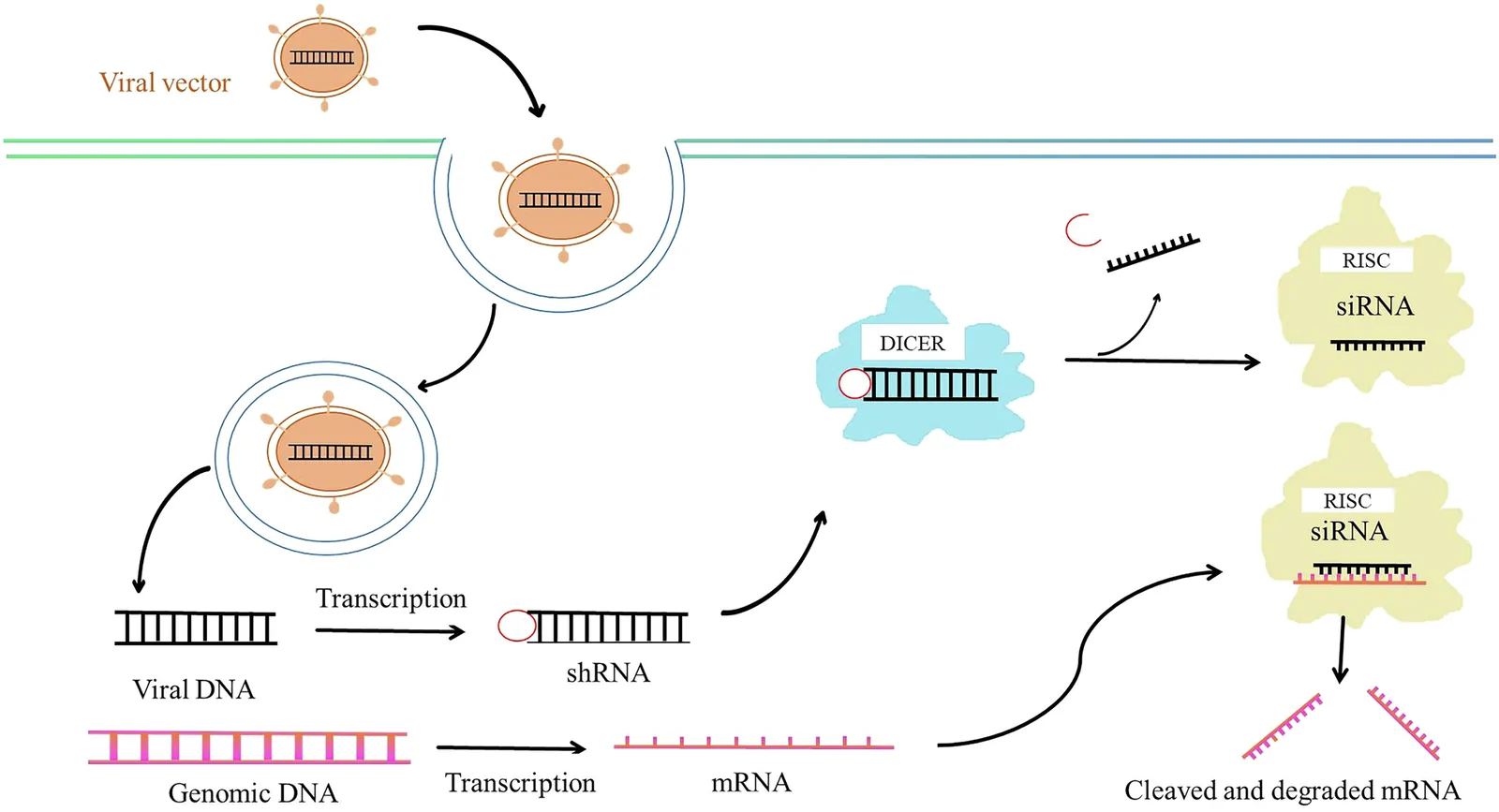
RNAi mechanism via viral vectors in plants. Mechanism of RNA interference (RNAi) mediated by viral vector-delivered shRNA in plant cells.

In the field of plant biotechnology, plant viruses as gene delivery vehicles have revealed potential for functional genomics, gene silencing, and temporary expression. It has allowed foreign genes to be transferred through plant viruses since they have a natural ability to infect plants and have an efficacious distribution throughout plant tissue. Since plant viruses are non-integrating, they can be an extremely useful tool when stable transformation is desired or unavailable^30^. Intermediaries derived from TMV, PVX, and CPMV^31^. Each virus has distinct benefits in terms of host range, systemic mobility, and the length of the inserted genomic regions^32^. Plant viruses are probably best known for their capacity to carry genomic sequences into many plants without the requirement for tissue culture, a time-consuming and genotype-dependent step associated with the most regularly employed methods of conventional transformation^33,34^^.^ For example, TMV and TMV-based vectors have established a successful system to express heterologous proteins, including both industrial enzymes and medicinal proteins, due to their rapid expression and scalability^35^. In addition, replicon-based systems that utilise geminiviruses like ACMV or BeYDV to replicate transgenes in the nuclei of plant cells in high copy numbers have also been developed. Gene silencing-based Techniques, such as VIGS, involving the integration of sections of the target genes into the viral genome to induce RNA interference (RNAi), are likely most successful with RNA viruses^25^. VIGS also offers speed and enables high-throughput functional assays of plant genes, especially in complex genome species or species with limited transformation tools (Fig. 1). Overall, plant-virus-mediated gene delivery is a flexible and effective tool for biotechnological purposes ranging from fundamental research to commercial-scale production of recombinant proteins^36,37^.

### Viral vectors for plant transformation

**Fig. 2:**
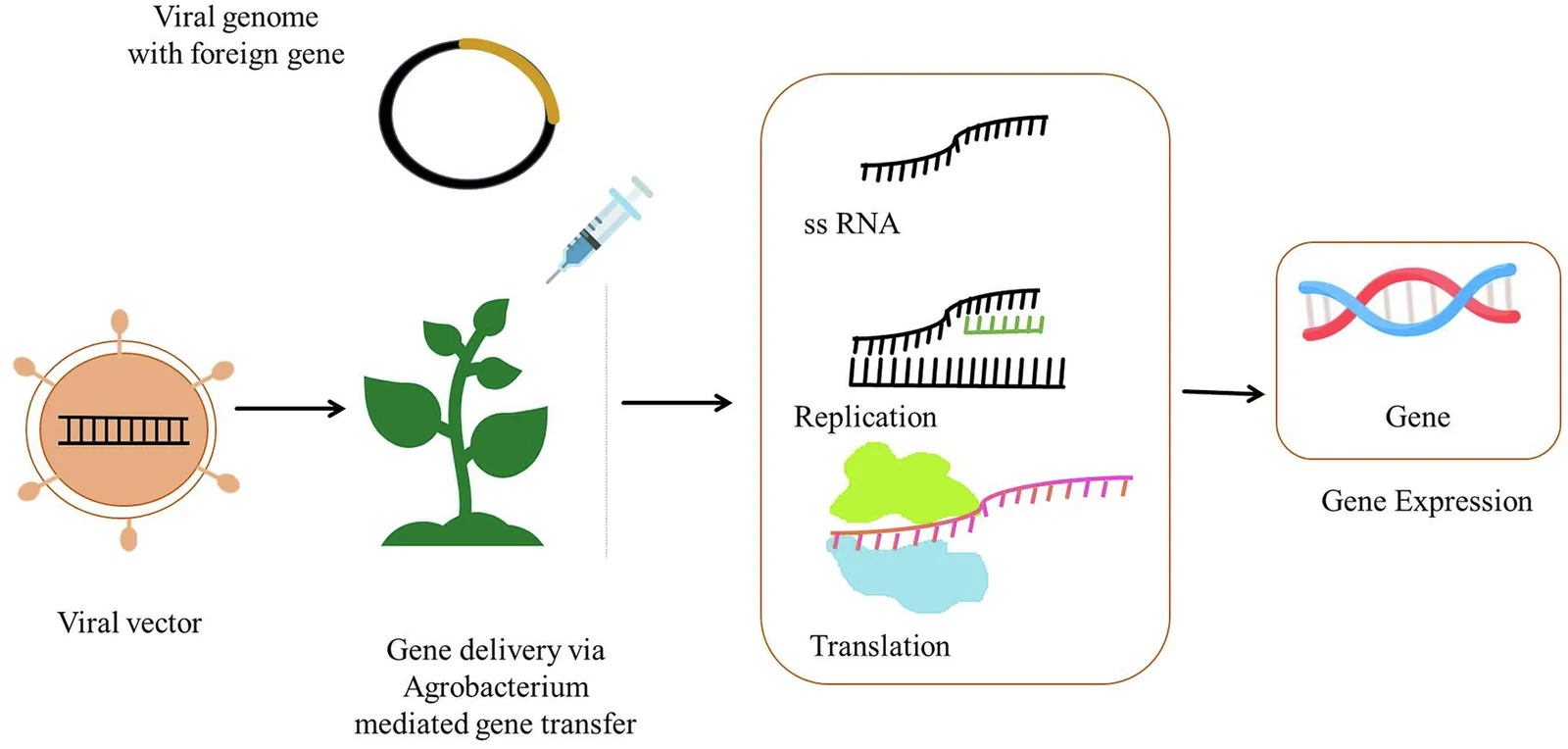
Plant virus-mediated gene delivery system. Diagrammatic illustrations of a gene delivery system and expression facilitated by plant viruses.

Plant viruses have evolved into stable transformation vectors for nuclear and plastid genomes, beyond the transient expression stage^38^. Traditional Agrobacterium-mediated transformation remains the best practice, but viral vectors have certain benefits, including not going through somatic embryogenesis and significantly reducing the time for genetic modifications. The two main categories of viral vectors include: replicative and non-replicative viral vectors. While non-replicative viral vectors are typically employed to transport gene editors, such as CRISPR-Cas components, replicative viral vectors are beneficial because they increase the target transgenes in plant cells. Geminivirus replicons, for instance, have been altered to promote a more effective HDR^39,40^. Recent studies have engineered viral vectors to deliver CRISPR/Cas9 and base editing systems, enabling precise genome editing in planta (Fig. 2). For example, TMV- and BeYDV-based vectors have been used for multiplex genome editing in *Nicotiana benthamiana*, rice, and tomato^41^ Furthermore, some vectors, such as BSMV and Foxtail mosaic virus (FoMV), have been used in monocot crops, broadening the utility of viral vectors for cereal improvements^42^. The versatility of viral vectors and their applications will continue to diversify through the use of synthetic biology protocols that allow for modularisation, tissue-specific promoters, and conditional expression systems^43,44^. Despite advances in vector technology, there are still limitations that arise, including cargo size limits that many RNA viruses have and the lack of stability of recombinant genomes. To address the limitations that arise from small RNA viruses, researchers are working on deconstructed viral vectors, where replication and movement functions are separated, allowing more flexibility and stability of vector systems. Thus, viral vectors provide a rapid, scalable, and customizable platform for plant genetic engineering, offering alternatives to conventional breeding and transgenic approaches (Table 1).

**Table 1:** Representative plant viruses used in biotechnology applications

Virus name	Genome type	Host range	Applications	Features	References
Tobacco mosaic virus (TMV)	( + )ssRNA	Broad (esp. Solanaceae)	Gene delivery, vaccine production, nano scaffolds	Stable rod-shaped particles; easily engineered	^41^
Potato virus X (PVX)	( + )ssRNA	Solanaceae	Gene expression, VIGS	Flexible filamentous virus; high expression	^31^
Cowpea mosaic virus (CPMV)	( + )ssRNA (bipartite)	Legumes	Nanotechnology, biosensors, and delivery	Icosahedral capsid; highly stable particles	^31^
Cucumber mosaic virus (CMV)	( + )ssRNA (tripartite)	Broad	Functional genomics, resistance studies	Can express foreign genes via RNA 3	^214^
Cauliflower mosaic virus (CaMV)	dsDNA (pararetrovirus)	Brassicaceae	Transgene expression (35S promoter)	Widely used 35S promoter from CaMV	^25,215,216^
Bean yellow dwarf virus (BeYDV)	ssDNA (geminivirus)	Legumes	DNA viral vectors for plant transformation	Enables replication and high expression in nuclei	^25^
Barley stripe mosaic virus (BSMV)	( + )ssRNA	Cereals	VIGS in monocots	One of the few tools for gene function in cereals	^42^
Peanut clump virus (PCV)	dsRNA	Monocots	Gene silencing studies	Natural silencing-inducing capacity	^217^
Tomato yellow leaf curl virus (TYLCV)	ssDNA (geminivirus)	Solanaceae	DNA vector for stable/transient expression	Used in CRISPR delivery and gene editing	^53^

A critical comparison of viral vector systems reveals trade-offs essential for selecting the right tool for a specific application. RNA virus vectors (e.g., TMV, PVX) are excellent for high-level, transient expression of proteins or VIGS due to their rapid replication^24,35^. However, their main bottleneck is a limited cargo capacity (typically <1–2 kb) and the genetic instability of larger inserts, which are often deleted during replication^45^. In contrast, DNA virus-based replicons (e.g., geminiviruses like BeYDV) can carry larger DNA fragments (up to several kb) and replicate to high copy numbers, making them suitable for delivering complex CRISPR machinery or large genomic sequences for gene editing via HDR^25,46^. However, they are confined to the nucleus and do not move systemically as efficiently as some RNA viruses^47^. For systemic delivery in cereals, vectors like BSMV and FoMV are among the few options available, but they are often limited to gene silencing rather than stable protein expression^42,48^. Mature technologies like TMV and PVX vectors are widely used in research and molecular farming^10^. In contrast, systems for delivering gene editors (e.g., CRISPR/Cas9 via geminiviruses) are still largely in the experimental phase, with key bottlenecks being the efficiency of delivery into plant germline cells and the risk of off-target effects^41,49^. The primary challenge for all these systems in moving from the lab to the field is the scalability of production and the development of robust containment strategies to prevent environmental spread^50^.

### Development of virus-resistant plants

As the worldwide burden of plant viral infections grows, producing virus-resistant crops becomes more important in terms of food security. Transgenic and non-transgenic biotechnological techniques for virus resistance are distinct. Pathogen-derived resistance (PDR) is among the oldest strategies, where viral genes such as coat protein (CP) or replicase were expressed in transgenic plants for viral reproduction prevention^51^. As an example, CP-mediated resistance has been used to successfully protect papaya against Papaya ringspot virus and squash against Zucchini yellow mosaic virus^35^. RNA-based techniques, particularly RNAi, have changed everything by allowing virus-resistant plants to be produced. Resistance is achieved by down-regulating crucial viral genes in a post-transcriptional manner using either hairpin RNA constructions or artificial microRNAs^52^. Authors have described RNAi-based resistance in tomato (*Solanum lycopersicum*) from Tomato yellow leaf curl virus (TYLCV) infection, and Rice (*Oryza sativa*) from infection with Rice tungro bacilliform virus, an RNA virus^53^ More recently, CRISPR-Cas9 and CRISPR-Cas13a genome editing have been used to develop virus-resistant plants through direct targeting of the viral genome (mainly DNA viruses), or that knocked out the factors contributing to vulnerability in a host^54^. For example, reducing the concentration of eIF4E, a host component necessary for potyvirus replication, can allow for either melon (*Cucumis melo*) or Arabidopsis (*Arabidopsis thaliana*) to be resistant^55^. Engineering synthetic resistance genes, as well as a decoy technique for trapping viral RNA silencing suppressors, is an emerging field. Furthermore, spray-induced gene silencing, in which double-stranded RNA is efficiently sprayed onto plant surfaces, offers a non-transgenic, environmentally friendly technique for viral control^44^. Despite these advances, the evolution of viral quasi-species and the emergence of resistance-breaking strains remain a concern. Thus, stacking resistance genes and using multiple defence mechanisms in combination is recommended for durable resistance.

## Applications of PVNPs in biotechnology

In nanobiotechnology, PVNPs have emerged as a versatile and promising family of nanomaterials, offering a unique combination of structural homogeneity, monodispersity, and biocompatibility. Since they do not productively replicate in mammalian cells, these nanoparticles prepared from the capsids of plant viruses such as TMV, CPMV, and PVX are ideal for a variety of biotechnological applications. They are applied in medicine for drug delivery, immunotherapy, diagnostics, vaccine production, bioimaging, and nanofabrication due to their ability to self-assemble, encapsulate, and be chemically or genetically modified^31,56,57^. This section points out the promise of PVNPs to revolutionise medical and other applications based on nanotechnology, and it also provides an extensive overview of the numerous applications.

### Biomedical applications

Biomedicine has been the major beneficiary of PVNPs, which are recognised as PVNPs for their extraordinary blend of being of natural origin, having a dimension at the nanoscale, being non-toxic and having surface chemistry that is chemically adjustable. Moreover, compared to engineered nanoparticles, PVNPs do not accumulate in tissues and are biodegradable by nature. These are excellent platforms for biological research because of their self-assembling nature, repetitive architecture, and structural homogeneity. Their areas of utilisation are gene editing devices, the next generation of cancer treatment methods, vaccines, and targeted drug delivery systems. Such uses rely on the fact that PVNPs are biologically inert in humans, and at the same time, they can be immune system modulators or delivery vehicles for highly targeted therapies^11,58^.

#### Drug delivery systems

Nanoscale drug delivery systems are the major sources of public interest for the use of PVNPs in medicine. In general, conventional drug delivery systems have a low concentration of the drug at the affected area, low bioavailability, and nonspecific toxicity. Payloads loaded in PVNPs lead to achieving one supply goal, better control of drug distribution and convenient surface functionalization to face the aforementioned challenges^59^.

##### Surface modification for targeting

Besides the chemical method, PVNPs can also be genetically modified to create those ligands, antibodies, aptamers, or peptides that will be able to bind specifically to receptors on the surface of ill cells^60^. For example, RGD peptides have been fused with TMV and CPMV to facilitate binding with the integrins that are overexpressed in cancer cells to promote tumour homing^59,61,62^.

- **Encapsulation and conjugation strategies:** PVNPs provide the option of drug conjugation that connects the drug to the exterior surface of the particle, as well as encapsulation, which refers to the drug loading inside the capsid. A drug such as doxorubicin or cisplatin in small molecular forms can be enclosed within the core channel of TMV, hence, avoiding both clearance from the blood and enzymatic degradation. The external surface can be functionalized to increase uptake by endocytosis or to protect the nanoparticle from immune recognition (e.g., PEGylation)^63^. For instance, doxorubicin conjugates with TMV: according to the research, doxorubicin conjugated with TMV is effective in treating triple-negative breast cancer^64^. The TMV-dox conjugates displayed the benefits of less cardiotoxicity, more tumour accumulation, and longer circulation compared to free drug products.
- **Overcoming biological barriers:** The blood-brain barrier (BBB) is the main obstacle for the treatment of brain diseases; however, it can still be crossed by PVNPs. TMV and PVX can be used for the treatment of glioblastoma^65^ or Alzheimer’s disease^66^ after being modified to deliver drugs via receptor-mediated transcytosis, enabling them to cross the BBB.
- **Controlled and stimuli-responsive release:** The next stage of PVNPs with a higher degree of control for the release of a drug in response to a redox gradient, pH, or enzymatic activity in diseased tissues is being developed. As an example, PVNPs have been decorated with pH-sensitive linkers that enable the release of the drug only in the acidic tumour tissue environment^64^.

#### Vaccine platforms

Vaccination was greatly changed with the introduction of VLPs, which are non-replicating, non-infectious particles that closely resemble native viruses and hence trigger strong immune responses. PVNPs are just the perfect match to be used in the creation of subunit vaccines, as they are the very definition of such vaccines.

- **Mechanism of immune activation:** Their repeating surface patterns that are PAMP-like allow PVNPs to be extremely immunogenic. The pattern of dendritic cells and macrophages is more efficient for uptake, thus antigen presentation through MHC molecules and generation of both humoral and cell-mediated immune responses occur^18^
- **Versatility in antigen display:** Genetically engineered plant parasites can be tailored to display human or animal disease antigens either through conjugation on the capsid surface or via targeted insertion into the coat-forming proteins. Consequently, by enabling the multivalent presentation of epitopes, the immunogenicity and B cell receptor crosslinking are enhanced^3^
- Case Studies:
  ○ **CPMV-SARS-CoV-2 vaccine:** One of the first steps in response to the COVID-19 pandemic, researchers modified CPMV particles to display receptor-binding domain (RBD) epitopes of the SARS-CoV-2 spike protein. The results in mice of preclinical studies showed excellent neutralising antibody titers and protection against the virus^67^ PVX- and TEV-derived virus-like nanoparticles (VNPs) have been functionalized with two distinct nanobodies, each designed to recognise separate epitopes within the RBD of the SARS-CoV-2 Spike protein^68^. Similarly,^69^ reported the development of a TMV-based nanoparticle vaccine candidate that presents the SARS-CoV-2 RBD derived from the viral Spike (S) glycoprotein. This RBD is essential for interaction with the human ACE2 receptor and represents a critical target for neutralising antibody responses^70^.
  ○ **PVX-HIV vaccine:** In vivo studies, PVX decorated with different conserved HIV epitopes to induce broadly neutralising antibodies, hence providing the possibility of overcoming the antigenic diversity of HIV^71^.
- **Edible vaccines:** Currently, oral vaccines are being developed with the help of TMV and other plant viruses expressed in food crops such as potatoes and tomatoes. It is an ideal vaccine for poor areas without the use of syringes and refrigeration^72,73^.
- **Thermal stability and production advantages**: most protein-based vaccines are sensitive to high temperatures; thus, their PVNP counterparts’ thermostability makes them easier to store and transport without any special conditions, as they can endure temperatures from 60 to 70 °C by themselves. The use of the transient expression method for the large-scale production of PVNPs in plants (e.g., *Nicotiana benthamiana*) allows for the production to be scaled up and be cost effective^74^.
- **Regulatory pathways and human trials**: along with excellent safety profiles, some PVNP-based vaccines have advanced to the stage of preclinical and first-phase clinical trials. The regulatory approval process for genetically engineered plant-derived biosafety VLPs is getting better with the relaxation of limitations as more data on biosafety are available^19^.

#### Cancer immunotherapy

Cancer immunotherapy is becoming one of the most rapidly expanding medical fields, and PVNPs are emerging as powerful immuno-oncology instruments. Given their capability to both promote innate and adaptive immunity, they can be used as tumour-targeting adjuvants, in situ vaccines, or immunostimulatory agent carriers, either alone or in combination with other agents^75^.

##### In situ vaccination and immune modulation

Vaccination as part of in situ immunotherapy necessitates the injection of immune-activating agents directly into the tumour mass. The use of CPMV has shown a great potential to reverse immunosuppressive tumour microenvironments, becoming in turn immunostimulatory through its own immune-stimulatory characteristics. CPMV, upon intratumoral injection, triggers immune cells’ Toll-like receptors (TLR2, TLR7, and TLR9). As a result, proinflammatory cytokines (IL-6, IFN-γ, and TNF-α) are secreted. The systemic anti-tumour immunity is turned on when activated dendritic cells and macrophages bring cytotoxic T lymphocytes to the site of antigen presentation^76^.

##### Combination therapies

The administration of PVNPs might enhance the efficacy of the immunotherapy by checkpoint inhibitors such as anti-PD-1 or anti-CTLA-4. For instance, the co-administration of CPMV together with the checkpoint inhibition drastically retarded tumour growth and prolonged the survival of melanoma-bearing mice. To potentiate further the effectiveness of the combination therapy, PVNPs are being examined not only for the targeted delivery of immunomodulatory agents such as cytokines, STING agonists, but also for the co-delivery of small-molecule immunomodulatory^77^.

- **Tumour tropism and penetration:** Although PVNPs such as TMV and PVX are not inherently tumour-killers, they can be re-targeted to some tumours that express the same receptors as by altering their surface properties (e.g., with folate, peptides, or antibodies). Their small size relative to larger particles makes it easy for them to penetrate tumours and release drugs in hypoxic or necrotic areas that are often inaccessible by traditional therapy^78^.
- **Personalisation and neoantigen display:** PVNPs will be utilised to display neoantigens in the future that refer to tumour antigens that are unique to an individual patient due to the mutations that occurred. To provide highly personalised cancer immunisations, these antigens might be delivered on PVNP carriers^79^.
- **Safety and immunogenicity:** PVNPs are by their nature non-replicative, non-cancerogenic, and non-infectious. In comparison to the live attenuated viral vectors used in other forms of immunotherapy, they are more secure since their systemic toxicity is very low, with a few studies confirming this^18^.

### Diagnostic and imaging applications

PVNPs have a lot of promise in medical imaging and diagnostics owing to their nanoscale accuracy, programmability, and biocompatibility. Among others, the technologies based on viruses enjoy the advantage that the multivalency may be used to increase the production of the signal and hence facilitate the detection of the biomolecules in even trace amounts. The precisely symmetrical structures of PVNPs allow them to be treated with molecular probes and labelling provided by several imaging techniques, which, in turn, make them visible with very high resolution in their diverse modalities. These features have enabled PVNPs to be used in various diagnostic systems, such as early detection of diseases, molecular imaging, and biosensors^80,81^.

#### Biosensing and detection platforms

Biosensors that use PVNP-based platforms are able to reach higher sensitivity, quicker detection, and higher specificity in such areas as pathogen detection, biomarker identification, and environmental monitoring. Because of their modularity, PVNPs can be decorated with targeted receptor molecules, such as aptamers, enzymes, antibodies, or nucleic acid probes, to make a new variety of sensing elements^82^.

- **Structural advantages for sensing:** The typical and precise layout of PVNPs, such as CPMV and TMV, is perfect for spatially ordered ligand presentation, which contributes to better performance of analyte binding. Through the use of the rod-like structure of TMV, enzymes or fluorophores have been consistently introduced along the capsid, the central channel being 4 nm wide and 300 nm long. This form of electrochemical or fluorescence sensors is likely to have higher signal-to-noise ratios^83^.
- **Electrochemical biosensors:** Electrochemical biosensors based on PVNP have been the source of eventually triumphant outputs in viral antigen as well as cancer biomarker sensing processes. To be specific, PSA ultra-high sensitive detection was made possible through electrodes modified with TMV. On the surface of TMV, scientists installed PSA-specific aptamers that made it possible to detect them at the picomolar level in clinical samples. Not only the large surface area of TMV but also excellent electron transport properties led to an electrochemical response facilitated^84^.
- **Colourimetric and optical sensors:** Plasmonic nanoparticles, which include quantum dots or gold nanoparticles (AuNPs), are one of the ways PVNPs can be used for improved optical signal generation. One of the most visible applications of CPMV immobilised with AuNPs is the recognition of nucleic acid sequences through the use of colourimetric and surface plasmon resonance (SPR) techniques for highly sensitive detection. These setups are ideal for point-of-care diagnostics as they are compatible with lateral flow assays that can be used in field conditions^85^.
- **Case study: SARS-CoV-2 detection:** PVNP-based biosensors were rapidly adapted to the COVID-19 pandemic. A biosensor of CPMV-AuNPs for the recognition of SARS-CoV-2 RNA sequences was recently reported. The biosensor proved the potential of PVNPs in portable diagnosis by showing low detection limits, rapid response times, and readout system compatibility with smartphones^68^.
- **Multiplexed detection:** PVNPs can interact with multiple types of materials at the same time because of their multivalent and modular characteristics. This property is handy in the case that a number of probe molecules have been fixed onto a single PVNP framework in multiplexed diagnostic applications. In the panel of inflammatory diseases, several cytokines can be recognised at the same time owing to fluorescence-based readouts utilising CPMV nanoparticles linked with different dyes^86^.

#### Molecular imaging

Medical imaging is very important in certain clinical areas, for example, in neurology, cardiology, and oncology. The biological compatibility of PVNPs, adjustable pharmacokinetics, and functional surface chemistry have enabled their use as contrast imaging agents for various imaging methods. Partly due to their regulated shape, the exact binding of imaging agents facilitates better resolution and localisation^64^.

- **Magnetic resonance imaging (MRI):** Contrast agents that contribute to the visibility difference between healthy and diseased tissues are essential for Magnetic Resonance Imaging (MRI). Gadolinium (Gd³⁺) or iron oxide nanoparticles have been modified to act as T1 or T2 contrast agents, respectively, in PVNPs such as the TMV and Papaya Mosaic Virus (PaMV). For instance, due to the slow rotational dynamics of the viral particle, TMV has been surface conjugated with Gd-DOTA complexes and has shown significantly higher T1 relaxivity than commonly used agents. Polyvalent metal nanoparticles (PVNPs) have been tagged with isotopes like ⁶⁴ Cu and Zr^84^ for positron emission tomography (PET) imaging^87^.
- **Positron emission tomography (PET)**: While the radioactive tags allow for sensitive imaging of metabolic or molecular activities, the surface ligands permit targeted delivery to neoplasms. It was very easy to image mice with breast tumors using CPMV conjugated with DOTA and labelled with ⁶⁴Cu due to the excellent tumor-to-background contrast that was attained. The molecules also hold promise in the conclusion of theranostics, the incorporation of diagnosis and treatment on the same platform^88^.
- **Optical and fluorescent imaging:** In the case of optical imaging, fluorescent groups like FITC, Alexa Fluor, and cyanine dyes may be attached to PVNPs. They are perfect for real-time imaging and surgical guidance due to their stability, which provides continuous fluorescence in vivo. For example, PVX has been modified with near-infrared dyes, which enable deep tissue penetration, for the monitoring of cancers in mice. Besides, these nanoparticles have a very low background autofluorescence that contributes to the clarity of images^89^.
- **Multimodal imaging:** One of the main advantages of PVNPs is that they can be compatible with multimodal imaging, which allows the seamless interaction of different channels to compensate for the individual weaknesses of single methodologies. For example, they allow combining MRI with PET or fluorescence. To make in-vivo anatomical imaging and functional imaging with perfect side effects null, TMV nanoparticles were designed that could carry Gd³⁺ and ⁶⁴Cu. These gadgets can be very productive in the medical fields for such applications as disease progression scoping, and treatments’ efficacies measurement^90^.
- **Targeting and biodistribution:** Usually, nanoparticle virus plant cell (PVNPs) is functionalized by cancer-targeting ligands like folate acid, transferrin, or peptide like iRGD for the enhanced targeting, which in turn will lead to less toxicity and higher selective accumulation of the targeted tissues. It is possible to adjust the PVNPs to be either with rapid renal clearance or with long circulation times according to pharmacokinetic studies based on the specific therapeutic requirements^91^.
- **Clinical translation and safety:** Imaginative agents based on PVNPs are undergoing clinical translation evaluation but are still in the preclinical stage. Meanwhile, research is progressing, and it keeps showing that they can be employed for a number of times without any danger because of their low toxicity, minimal immune response, and good elimination. PVNPs also have a few issues with biodegradability and long-term accumulation compared to synthetic nanoparticles such as liposomes or carbon nanotubes^92^.

### Agricultural and environmental applications

In contrast to their thorough study in biological fields, PVNPs still have major applications in environmental and agricultural sciences^93^. They are very attractive, particularly for soil quality, crop protection, bio-sensing, and decontamination, whose putative sustainability is due to their biodegradability, genetic engineering, and natural origin. PVNP-based methods deliver a new generation of devices to rejuvenate crop production and environmental tolerance without the heavy utilization of artificial agrochemicals, thus stepping in to save agriculture from various threats such as new diseases, global warming, and the incorrect use of chemicals^92,94^.

#### Nanopesticides and biocontrol agents

The indiscriminate and excessive use of chemical pesticides in modern agriculture has led to pesticide resistance, water and soil pollution, and pesticide toxicity to non-target organisms, among other environmental and health issues. Plant virus-based nanoparticles (PVNPs) with their wide-ranging effectiveness, lowered environmental impact, and modulated selectivity are gaining more and more recognition as green alternatives to targeted pest control and biocontrol^95,96^.

- **PVNPs as nanopesticide carriers:** TMV and PVX are viruses that can be modified to deliver insecticides, which can be either synthetic or natural, in a controlled manner and thus be used against a certain pest or disease. In detail, azadirachtin, a natural biopesticide that is derived from neem, has been encapsulated in PVNPs for the purpose of increasing its stability against UV radiation, ensuring controlled release, and leaf adhesion^97^.
- **Specificity and reduced off-target effects:** The availability of the surface modifiability of PVNPs makes it possible to direct them towards pest-specific receptors without hurting insects like bees that are helpful in pollination. As an illustration, by attaching ligands that are specific to the intestinal lining of Helicoverpa armigera caterpillars, TMV has been made capable of local toxin delivery after consumption. The needed pesticide dose becomes lower, and resistance development is limited through this targeted precision^98^.
- **Direct antimicrobial effects:** In some cases, plant viruses have been found to have antiviral, antibacterial, or antifungal effects over time. When healthy plants are pre-treated with CPMV, the latter may exert a direct antiviral effect against plant-pathogenic viruses, which is called cross-protection. At the same time, engineered TMV nanoparticles combining silver ions or antifungal peptides have a wide range of applications as they can be utilised to halt fungal growth, for example, Botrytis cinerea^99,100^.
- **Smart release formulations:** PVNPs can become components of stimulus-sensitive delivery systems where the active ingredients are released only when a certain variable, e.g., pH, temperature, or moisture, changes, factors that are very likely to be influenced by fungal invasion or insect feeding. Being able to reduce waste and extend field life, PVNPs are the key feature of precision agriculture^101^.
- **Environmental and regulatory benefits:** PVNP-based nanopesticides are those that can decompose in the soil completely without leaving any hazardous residues. Their safety to the environment, low toxicity to mammals, and low frequency of use are in line with the recommendations of the FAO Code of Conduct on Pesticide Management and the United Nations Sustainable Development Goals (e.g., SDG 2: Zero Hunger, SDG 12: Responsible Consumption and Production)^102^.

#### Delivery systems for genetic material in plants

The most critical technological hurdle in the enhancement of crops is the effective delivery of genes to plants, particularly in mature tissues and non-model organisms. The efficiency of these conventional methods, e.g., biolistics and Agrobacterium-mediated transformation, is quite low, and they also suffer from problems related to off-target effects and regulations; hence, they have some limitations. Recently, PVNPs have been widely recognised as a method that can overcome these challenges in plant genetic engineering as they provide a non-transgenic, transient, and scalable approach for the delivery of RNA, DNA, and CRISPR elements into plant cells^103^.

- **RNA interference (RNAi) via PVNPs:** Double-stranded RNA (dsRNA) or small interfering RNA (siRNA) has been handed over by PVNPs such as Cowpea chlorotic mottle virus (CCMV) and TMV to initiate gene silencing in the targeted pest or plant. VIGS is a fast and cost-effective method of plant disease defence and functional genomics. For instance, pest survival was lowered to a significant extent by TMV-based vectors, which made the expression of dsRNA targeting whitefly detoxifying enzymes. Another study was revealed during the field test where RNAi administration based on PVX could lessen crop destruction by knocking down worm effector genes^96^.
- **Genome editing and CRISPR delivery:** PVNPs have been demonstrated to carry Cas9 ribonucleoprotein complexes as well as gRNAs into plant cells for exact genome modifications at the targeted site without going through transgenic intermediates^104,105^. (Research in 2023 managed to transfer CRISPR/Cas configurations into Nicotiana plants using TMV nanoparticles, thus generating transient gene editing without the integration of exogenous DNA–a release of regulatory and biosafety perspectives^33^.
- **Multiplexed gene regulation:** The ability of PVNPs to carry multiple genetic payloads has made it possible to manipulate many genes simultaneously, thus turning genes on or off to produce multigene complex traits such as drought resistance, nutrient use efficiency, or pest resistance. These traits can be developed on a fast-track basis through this multiplexing, which not only shortens breeding cycles^106^.
- **Non-heritable, transient expression:** Gene delivery via PVNPs is one of the major advantages, as the alterations are usually ephemeral and untransmittable, which means they may not fall under the categorisation of GMOs in different legal areas. Besides, they are compatible with the breeding of the next generation^107^.

#### Environmental monitoring and remediation

Besides the main agricultural functions, PVNPs are also used for a new purpose: pollution remediation, biosensing, and environmental monitoring. They are ideal for green nanotechnology due to their structural stability, adaptability, and compatibility with various functional materials^108^.

- **Heavy metal detection:** Toxic heavy metals such as lead (Pb²⁺), mercury (Hg²⁺), and cadmium (Cd²⁺) in water and soil have been identified by PVNPs decorated with chelating ligands or fluorescent tags for functionalization. By way of example, thiol-peptide modified CPMV particles showed high selectivity and sensitivity towards Hg²⁺ in river water samples. These biosensors are perfect for environmental regulatory monitoring as they provide a fast on-site test with low detection levels (sub-ppb range)^109^.
- **Pesticide residue sensing:** Scientists have created biosensors that are capable of detecting organophosphate pesticides, which employ PVNPs as the base for enzymatic sensors (e.g., acetylcholinesterase). Even in complex agricultural runoff samples, TMV-based platforms maintain the sensitivity and stability of the sensor by catalytically immobilising enzymes on the large surface area provided^110^.
- **Soil health and microbiome modulation:** The latest discoveries have revealed that the use of PVNPs as delivery agents of health-promoting substances (e.g. probiotics or biofertilizers) can change the soil microbial communities. In order to recover rhizosphere health and encourage nutrient uptake in cereals and legumes, for example, TMV nanoparticles with nitrogen-fixing spores of bacteria or biofilm-stimulating peptides have been employed^111^.
- **Bioremediation agents:** The use of functionalized PVNPs might be extended to support applications or as nanocatalysts that are capable of completely removing pollutants (e.g., hydrocarbons, dyes, and insecticides) from contaminated environments. PVX, that is, iron oxide functionalized, has been applied in a Fenton-like process to discolour commercial dyes. The major source of groundwater pollution, which is trichloroethylene, has undergone the process of hydrogenation by TMV-templated nanowires, which are further functionalized with palladium^112^.

**Biodegradability and ecotoxicological safety:** Above all, PVNPs are safe for all animals, which are not the targeted species, as they break down in nature into non-toxic peptides or nucleic acids. An opposite low permanence characteristic is observed in synthetic nanomaterials that may concentrate in ecosystems and pose a threat to aquatic and soil biota^113^.

## Essential bioinformatics tools for advancing viral research

Viruses constitute one of the most varied groups of biological entities on the planet, collectively forming what is commonly known as the virosphere. It is estimated that there are around 10³¹ viral particles in existence worldwide, and for billions of years, their ongoing replication and mutation have resulted in an unmatched reservoir of genomic diversity. The mutation rates differ considerably: DNA viruses generally have rates ranging from 10⁻⁸ to 10⁻⁶ substitutions per nucleotide for each cell infection, while RNA viruses show higher rates between 10⁻⁶ and 10⁻⁴ substitutions. This vast genetic variability underscores the need for robust computational tools that can (i) identify viral sequences and functional elements, (ii) predict, annotate, and compare their biological functions, and (iii) organise data to convert raw measurements into significant biological insights^114,115^). Historically, the understanding of viruses has been restricted to a limited array of well-explored isolates, yet viral infections continue to pose a significant threat to human health, agriculture, and animal husbandry. Traditional diagnostic techniques, including microscopy, antigen detection assays (like ELISA), and molecular methods such as PCR, qPCR, and RT-qPCR, have demonstrated high sensitivity in identifying specific pathogens and quantifying viral loads. However, these methods are limited to detecting only the viral sequences that closely align with pre-designed primers or probes; as a result, infections from rare variants or completely new viruses often remain undetected^116,117^. Advancements in bioinformatics for primer design have broadened the diagnostic capabilities of PCR-based assays, yet these approaches still inherently rely on known viral diversity. To address these constraints, untargeted approaches like shotgun sequencing and metagenomic analysis are being increasingly utilised to obtain viral genetic material without prior knowledge of its identity^116,118^.

The last few decades have seen swift technological progress from the emergence of high-throughput sequencing (HTS) and mass spectrometry to the incorporation of computational sciences, including bioinformatics, ML, and systems-level “omics” strategies. Bioinformatics has become essential to viral research, offering specialised tools for the analysis of vast and intricate datasets. Basic applications involve quality assessment of NGS data, aligning nucleotide or protein sequences, and conducting statistical tests. More sophisticated applications encompass genome annotation pipelines, metagenomic profiling, and studies linking genotype to phenotype^114,115^. Crucially, these bioinformatics methods have revolutionised viral taxonomy. With the support of computational classification techniques, the number of recognised viral lineages has significantly increased. The ICTV has recently updated the hierarchical framework for viral classification, now acknowledging 15 taxonomic ranks: realm, subrealm, kingdom, subkingdom, phylum, subphylum, class, subclass, order, suborder, family, subfamily, genus, subgenus, and species (Table 2). This more detailed classification offers a broader structure for organising viral diversity and highlights the essential role of bioinformatics in contemporary virology^119^.

**Table 2:** List of computational software and tools used for virus taxonomy

S. no.	Name of Tool/software/method	Working	Input	Output	Advantages	Disadvantages	Important thresholds	References
1	CAT (Contig Annotation Tool)	Classifies sequences taxonomically using sequence alignments	Contigs or reads	Taxonomic assignments	High accuracy, genome-level resolution	Computationally intensive for large datasets	Default e-value < 1e-5	^218^
2	Kraken	K-mer based taxonomic classification	Reads or contigs	Taxonomic labels	Very fast, scalable	Requires large reference database	Confidence score default 0.1	^219^
3	KrakenUniq	Variant of Kraken using unique k-mers	Reads or contigs	Taxonomic labels with abundance	Reduces false positives, quantitative	Large memory usage	Default thresholds as Kraken	^220^
4	ClARK	K-mer based classifier for microbial/viral reads	Reads	Taxonomic assignment	High speed, scalable	Less sensitive for highly divergent sequences	Confidence threshold adjustable	^221^
5	MetaCache	K-mer based taxonomic profiling	Reads	Taxonomic profiles	Fast and memory efficient	Limited to reference database coverage	Default k-mer 31	^222^
6	Ganon	Taxonomic classification using k-mer indexing	Reads	Taxonomic labels	Efficient indexing, scalable	Database-dependent	Adjustable classification thresholds	^223^
7	VirTool	Web-based virus detection and taxonomy	Reads	Virus identification and classification	Integrates multiple tools, easy to use	Limited offline usage	Depends on built-in modules	^224^
8	VirFinder	Machine learning based virus detection	Reads	Predicted viral sequences	Can detect novel viruses	Requires training data	Score threshold default 0.9	^159^
9	VirSorter	Viral sequence identification using reference and HMM	Contigs	Viral contigs and confidence scores	Identifies novel viruses	False positives in complex metagenomes	Score threshold categories 1–3	^118^
10	VirSorter2	Improved version of VirSorter	Contigs	Predicted viral sequences	Better sensitivity, integrates multiple models	Still false positives possible	Default confidence thresholds	^225^
11	DeepVirFinder	Deep learning-based viral detection	Reads/contigs	Predicted viral sequences	Detects highly divergent viruses	Computationally intensive	Score threshold adjustable	^159^
12	GRAViTy	Viral genome-based taxonomy assignment	Viral genomes	Taxonomic classification	Accurate phylogenetic placement	Requires complete or near-complete genomes	-	^130^
13	vConTACT	Network-based viral classification	Viral genomes	Taxonomic clusters	Captures evolutionary relationships	Limited by database completeness	Default clustering parameters	^129^
14	VICTOR	Genome-BLAST distance phylogeny for viruses	Viral genomes	Taxonomic tree	High-resolution viral taxonomy	Requires complete genomes	Default distance thresholds	^140^
15	DEmARC	Distance-based viral taxonomy	Viral genomes	Classification and clustering	High-resolution taxonomic assignment	Computationally intensive	Cut-off thresholds defined in method	^139^
16	PASC (Pairwise Sequence Comparison)	Compares nucleotide or amino acid sequences pairwise to estimate similarity.	Viral nucleotide/protein sequences	Percent identity scores for classification	Simple, intuitive; widely used in ICTV classification	Limited resolution for divergent sequences	70–85% for species demarcation	^137^
17	PhyloPlace	Places query sequences into reference phylogenetic trees using maximum likelihood.	Viral genome/sequence data	Phylogenetic placement of sequences	Efficient for large datasets; scalable	Dependent on high-quality reference trees	Placement score thresholds	^226^
18	APCluster (Affinity Propagation Clustering)	Clusters sequences using message-passing between data points without predefining cluster number.	Pairwise similarity/distance matrices	Clusters of related viruses	Does not require pre-set number of clusters; robust	Sensitive to input parameters; computationally heavy for very large datasets	Preference and damping factors	^227^
19	mPTP / VirTree	Uses multi-rate Poisson Tree Processes to infer species boundaries from phylogenetic trees.	Phylogenetic trees from sequence alignments	Delimited species clusters	Better species delimitation accuracy	Relies on tree quality; may over-split	Branch support values	^228^
20	CASTOR	Machine learning framework that classifies viral sequences based on codon usage and k-mer patterns.	Viral genomic sequences	Taxonomic classification of viruses	Captures genome composition features	Requires training datasets	Model cut-offs; accuracy metrics	^229^
21	PhyClip	Clusters viral sequences using phylogenetic branching and likelihood optimization.	Sequence alignments + phylogenetic trees	Clusters/taxonomic groupings	Flexible, statistically rigorous	May misclassify when branch lengths are uneven	*p* value/likelihood thresholds	^230^
22	Mycov	Specialized tool for mycovirus classification using similarity and phylogeny.	Mycovirus genome/protein sequences	Taxonomic placement of mycoviruses	Tailored for fungal viruses	Limited to mycoviruses; smaller database		^231^
23	VIRIDIC	Calculates pairwise intergenomic similarities to define species/genus boundaries.	Viral genome sequences	Intergenomic identity matrix & classification	Web-based, fast, ICTV-compliant	Limited to complete genomes	Species cut-off ~95%; genus cutoff ~70%	^232^

### Sequence analysis software

Modern computational resources are crucial for the analysis of viral sequences, aiding precise classification, genome assembly, and studies of evolution. The methodologies utilised to bolster viral taxonomy typically depend on four primary factors: (i) variations in nucleotide or amino acid patterns, (ii) similarities in genes or proteins, (iii) the reconstruction of phylogenetic trees, and (iv) measurements of pairwise genetic distances^114,118^. Although the selected method often hinges on the research goal and the specific host–virus interaction being examined, integrating these methods can provide stronger classifications that accurately depict evolutionary connections^119^.

**Fig. 3:**
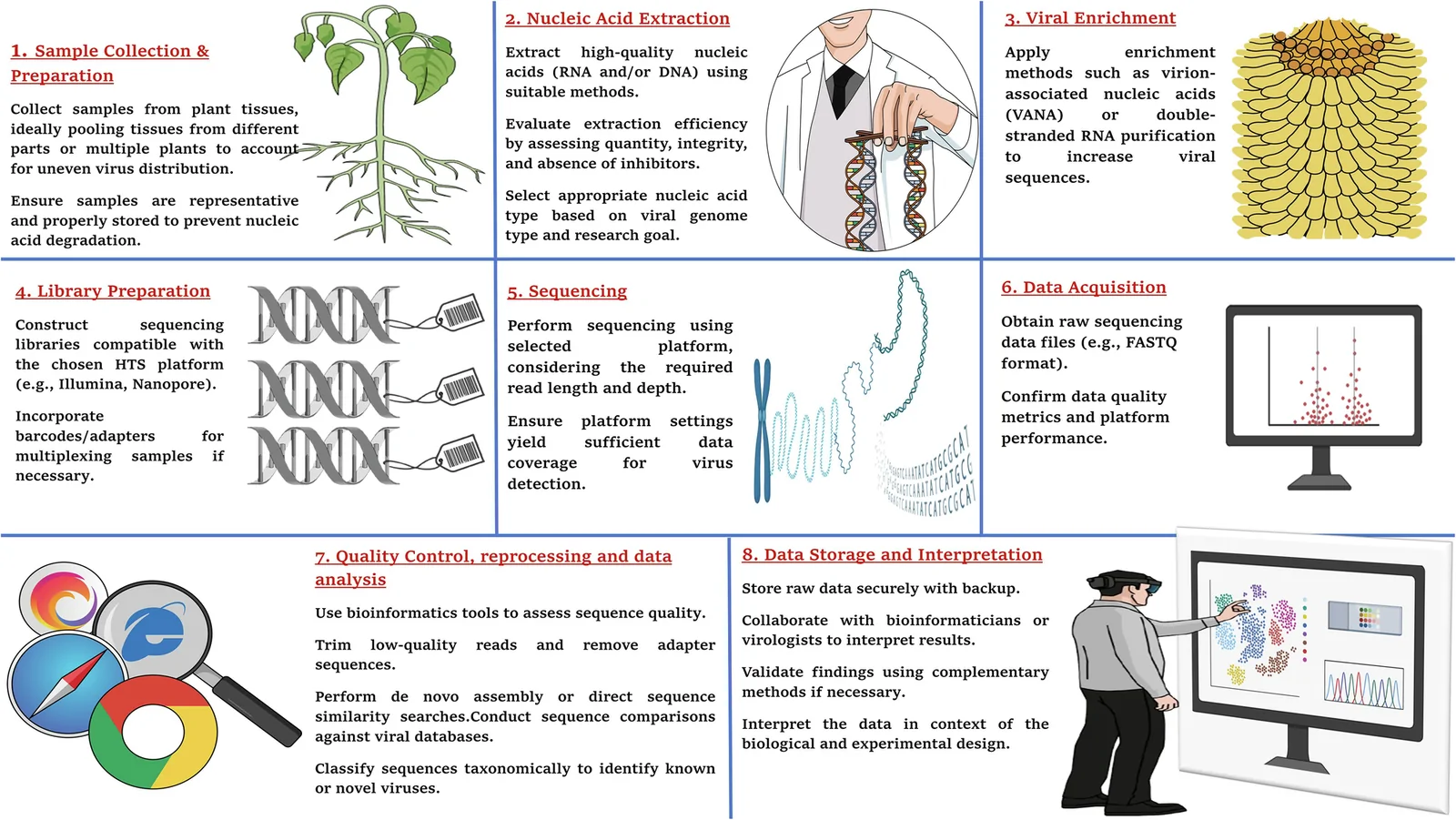
High-throughput sequencing workflow. Procedure of High Throughput sequencing: (1) Sample collection and preparation (2) Nucleic acid extraction (3) Virus enrichment (4) Genomic library preparation (5) Sequencing (6) Data Acquisition (7) Quality control and assessment of data (8) Data storage and interpretation

The most straightforward sequence-based techniques involve utilising statistical descriptors, such as overall nucleotide makeup (e.g., G + C content) or frequency profiles of k-mers, where all potential oligonucleotides or peptide sequences of a defined length are counted. These k-mer-based techniques have shown effectiveness in viral classification, but they can be influenced by various confounding elements, like uneven sampling, recombination, convergent evolution, or specific mutation rates in lineages. Additionally, interpreting variations from k-mer statistics can be complex, which often makes alignment-based strategies more informative (Table 3). The rise of HTS, encompassing both second-generation (short-read, massively parallel techniques) and third-generation (long-read, single-molecule methods), has transformed the field of viral genomics. Before downstream analyses, raw sequencing output is subjected to quality control procedures to eliminate sequencing errors (Fig. 3). Measures such as Phred quality scores gauge the likelihood of nucleotide errors, while heuristic filters identify lingering primers, adapters, overrepresented k-mers, and atypical GC content. Common tools utilised during this phase include FastQC and MultiQC^120,121^, alongside pre-processing applications like PRINSEQ^115^ that create straightforward reports to assist in pre-processing tasks. Subsequent trimming or error correction enhances read accuracy, thereby assuring reliable analyses downstream^116^. For characterising viral genomes, sequencing reads can be aligned with established reference genomes to pinpoint variants or uncover quasispecies; variant-calling tools subsequently assist in detecting sequence polymorphisms, guiding the creation of refined primers for PCR-based methods. If no closely related reference genome is available, de novo assembly becomes crucial. Millions of short reads are algorithmically joined to form contiguous sequences (contigs), and specific viral assemblers like VICUNA and IVA have been created to tackle assembly issues unique to viral genomes^114^. Two primary types of HTS platforms dominate viral research ^122^:

- **Short-read HTS (NGS):** represented by Illumina, which generates millions of reads comprising several hundred nucleotides with minimal error rates.
- **Long-read HTS (single-molecule sequencing, SMS):** represented by Pacific Biosciences (PacBio) (Rhoads & Au, 2015) and Oxford Nanopore Technologies (ONT), producing reads that cover tens to hundreds of kilobases, enabling the capture of complete viral comprehensive potential of HTS for viral discoverygenomes in a single read^123,124^.

**Table 3:** List of computational softwares and tools used for sequence alignment

S. no.	Name of tool/software/method	Working	Input	Output	Advantages	Disadvantages	Important thresholds	References
1	FastQC	Quality control for high throughput sequence data	Raw reads	Quality reports	Easy to use, comprehensive metrics	Limited to raw data, no trimming	-	^120^
2	PRINSEQ	Preprocessing of sequence data	Raw reads	Processed reads	Removes low-quality bases, adapters	May discard useful data if thresholds are too strict	Quality score threshold adjustable	^233^
3	MultiQC	Aggregates results from multiple QC tools	QC reports	Combined summary report	Visualizes data from multiple sources	Relies on outputs from other tools	-	^121^
4	Poretools	Analysis of Oxford Nanopore sequencing data	Raw nanopore reads	Processed data	Handles long reads efficiently	Limited support for other platforms	-	^123^
5	NanoOK	Quality control for Nanopore reads	Raw nanopore reads	Quality metrics	Specifically designed for Nanopore data	Limited to Nanopore data	-	^234^
6	Cutadapt	Removes adapter sequences from reads	Raw reads	Adapter-free reads	Efficient and widely used	May not detect all adapter types	Adapter sequence threshold adjustable	^235^
7	Trimmomatic	Trims Illumina reads	Raw reads	Trimmed reads	Handles paired-end data well	May require tuning for optimal performance	Quality score threshold adjustable	^236^
8	BBduk	K-mer based adapter and quality trimming	Raw reads	Trimmed reads	Handles various trimming tasks	May be slower for large datasets	K-mer size threshold adjustable	^116^
9	NanoCorrect	Corrects errors in Nanopore reads	Raw Nanopore reads	Corrected reads	Improves accuracy of long reads	Computationally intensive	-	^237^
10	PoreSeq	Error correction for Nanopore sequencing	Raw Nanopore reads	Corrected reads	Enhances read accuracy	Requires high computational resources	-	^238^
12	NaS (Nanopore Synthetic Long Reads)	Nanopore sequencing error correction	Raw Nanopore reads	Corrected reads	Enhances read quality	May not handle all error types	-	^239^
13	DIAMOND	Performs fast protein sequence alignment using double indexing and optimized seed search algorithms. Designed as a faster alternative to BLAST.	Protein/DNA sequences (FASTA format)	Aligned sequences with similarity scores	Extremely fast (thousands of times faster than BLAST), scalable for large datasets, good sensitivity	Slightly reduced sensitivity compared to BLAST, optimized mainly for protein sequences	E-value (default 0.001), identity %, bit score	^240^
14	HMMER	Uses profile Hidden Markov Models (HMMs) to detect sequence homology and annotate protein domains. Highly sensitive for remote homology searches.	Protein/DNA sequences, profile HMM databases	Homology search results with E-values, domain annotations	Very sensitive for detecting remote homologs, widely used in Pfam/InterPro	Slower than heuristic tools like DIAMOND, computationally intensive on large datasets	E-value (default ≤ 0.01), bit score thresholds	^241^

Quality evaluation tools for long-read datasets include Poretools and NanoPack, with error correction achievable through either non-hybrid tools or hybrid approaches that merge short- and long-read information. Assembly algorithms are similarly categorised: non-hybrid assemblers (e.g., Canu, Miniasm) specialise in long-read data, while SPAdes and Unicycler bring together short- and long-read datasets to optimise accuracy^125–127^ Together, these sequence analysis tools create a solid basis for viral genomics, enabling precise genome reconstruction, variant identification, and phylogenetic classification. They continue to develop alongside sequencing technologies, creating exceptional opportunities for the exploration of the vast diversity found within the virosphere.

**Limitations of HTS**

1. **Infrastructure and expertise prerequisites**
  HTS produces extensive datasets that necessitate strong computational infrastructure and specialised knowledge for management and analysis. Many diagnostic and smaller laboratories do not have access to these resources, and the lack of trained bioinformaticians complicates implementation significantly.
2. **Expense of bioinformatics analysis**
  While the sequencing process itself demands considerable investment, data analysis often adds another financial strain, sometimes constituting ~15% of the overall project expenditure. This includes the costs associated with hiring skilled bioinformaticians and the infrastructure required for long-term data storage and maintenance.
3. **Software availability and limitations**
  Commercial sequence analysis tools with graphical user interfaces provide convenience and user-friendliness for standard tasks such as mapping and assembly. However, these tools can be prohibitively costly and may lack the flexibility needed for more specialised applications. Free “all-in-one” viral detection pipelines are available, but their functionality is typically limited to fundamental classification, and installation may require advanced computational skills.
4. **Command-line difficulties**
  Many bioinformatics programs are specifically tailored for Linux operating systems and can only be accessed through the command line. This necessitates familiarity with programming languages such as Shell, Python, or R, making these tools less accessible to researchers lacking computational training.
5. **Challenges in interpretation**
  Interpreting sequencing outcomes goes beyond computational analysis. To derive meaningful biological insights, it is essential to foster collaboration between bioinformaticians and plant virologists, or have plant virologists who are well-versed in bioinformatics.
6. **Data storage and management**
  HTS initiatives generate substantial quantities of raw data, frequently amounting to hundreds of gigabases per sequencing run. For example, the Illumina NextSeq system can yield between 120 and 300 gigabases per run, resulting in file sizes that range from 39 to 170 GB. Ensuring adequate storage, secure backup, and long-term data preservation typically exceeds the capabilities of standard laboratory systems. While cloud computing and cluster-based solutions like Galaxy provide valuable alternatives, they require high-speed internet access and may incur additional expenses^122^.
7. **Sequencing error rates**
  Short-read sequencing techniques are generally very accurate but frequently encounter challenges with repetitive or structurally complex genomic regions. In contrast, long-read sequencing can cover entire genomic fragments but tends to have higher error rates, which necessitates hybrid strategies that integrate both approaches to enhance assembly precision.
8. **Reliance on reference databases**
  The effectiveness of taxonomic classification is heavily reliant on the thoroughness of available reference databases. If novel or significantly divergent viruses are not included in existing repositories, they may be overlooked or incorrectly classified, which limits the comprehensive potential of HTS for viral discovery.

### Comparative genomics

Comparative genomics serves as a crucial approach in the field of viral research, enabling scientists to examine, compare, and interpret genomic data across various viral species. This method’s strength lies in its capacity to yield insights into viral diversity, genome structure, evolutionary lineage, and the interactions between hosts and viruses. In contrast to traditional virology methods that typically depend on virus isolation and culture, comparative genomics leverages HTS and metagenomic techniques to directly identify viral genomes from environmental or biological samples (Table 4). This has broadened the scope of viral discovery by revealing previously unknown and unculturable viruses, often represented as uncultivated virus genomes (UViGs)^128^. To facilitate the effective sharing and comparison of these genomic resources, guidelines like the Minimum Information about an Uncultivated Virus Genome (MIUViG) have been developed to standardise metadata and annotation practices^118^.

**Table 4:** List of computational softwares and tools used for genomics and proteomics

S.No.	Name of tool/software/method	Working	Input	Output	Advantages	Disadvantages	Important thresholds	References
1	SPAdes	Assembler for single-cell and multi-cell data	Reads	Assembled contigs	High-quality assemblies	May struggle with highly repetitive regions	-	^242^
2	Canu	Assembler for long reads	Long reads	Assembled contigs	Optimized for long reads	High memory usage	-	^243^
3	Miniasm	Rapid assembler for long reads	Long reads	Assembled contigs	Fast assembly process	May produce fragmented assemblies	-	^244^
4	Unicycler	Hybrid assembler for Illumina and Nanopore data	Illumina and Nanopore reads	Assembled contigs	Combines strengths of both platforms	Computationally intensive	-	^245^
5	MEGAHIT	Assembler for large and complex metagenomes	Reads	Assembled contigs	Efficient for large datasets	May not handle highly repetitive regions well	-	^244^
6	metaSPAdes	Metagenomic assembler	Metagenomic reads	Assembled contigs	Optimized for metagenomic data	High memory usage	-	^246^
7	Ray-meta	Metagenomic assembler	Metagenomic reads	Assembled contigs	Scalable and efficient	May produce fragmented assemblies	-	^247^
8	IBDA-UD	Assembler for ultra-deep metagenomic data	Metagenomic reads	Assembled contigs	Handles ultra-deep sequencing data	High computational requirements	-	^248^
9	BWA	Short read aligner	Reads	Aligned sequences	Fast and accurate	Limited to short reads	-	^142^
10	Bowtie	Ultrafast aligner for short reads	Reads	Aligned sequences	Very fast alignment	Limited to short reads	-	^249^
11	BBmap	Aligns reads to reference genomes	Reads	Aligned sequences	Handles various read types	May be slower for large datasets	-	^116^
12	STAR	Spliced aligner for RNA-seq data	RNA-seq reads	Aligned sequences	Handles spliced alignments well	High memory usage	-	^250^
13	GraphMap	Long read aligner	Long reads	Aligned sequences	Optimized for long reads	May not handle short reads well	-	^251^
15	ORF Finder	Identifies open reading frames	DNA sequences	Predicted ORFs	Easy to use, web-based	Limited to simple sequences	-	^252,253^
16	Prodigal	Gene prediction tool	DNA sequences	Predicted genes	Accurate and fast	May miss some genes in complex genomes	-	-
17	VIGOR	Gene prediction tool	DNA sequences	Predicted genes	Handles viral genomes well	Limited to viral genomes	-	^55^
18	BLAST	Sequence alignment tool	Sequences	Alignment results	Widely used, comprehensive database	May miss distant homologs	E-value threshold adjustable	^116^
19	HHpred	Protein structure prediction	Protein sequences	Predicted structures	High accuracy, comprehensive database	Computationally intensive	-	^117^
20	V-PIPE	An automated pipeline for analyzing viral NGS data; supports assembly, alignment, variant calling.	Raw sequencing reads (FASTQ)	Consensus genomes, variants, alignments	End-to-end workflow, reproducible, adaptable	Computationally intensive, requires installation	Quality score thresholds for variant calling	^254^
21	pVSUPPred	Machine learning method for predicting virus–host protein–protein interactions.	Viral and host protein sequences	Predicted PPI interactions	Helps understand virus–host interactions, useful for drug target discovery	Predictions depend on training data quality	Confidence score cutoffs	^255^
22	VirFinder	K-mer based machine learning classifier to identify viral sequences from metagenomic data.	Metagenomic sequences	Probability scores of viral origin	Fast, alignment-free	May misclassify short sequences	*P*-value < 0.05 indicates viral sequence	^256^
23	VirusDetect	NGS-based pipeline for detecting viruses and viroids in plants.	Plant small RNA or transcriptome data	Virus/viroid detection reports, contigs	Specialized for plant viruses, integrates de novo assembly	Requires good sequencing depth	Coverage depth threshold	^257^
24	VirTool	Web-based platform for virus identification and annotation from high-throughput data.	NGS data	Virus identification and annotation	User-friendly, web accessible	Limited to supported virus databases	E-value cutoffs, coverage %	^258^
25	VirAnnot	Bioinformatics tool for functional annotation of viral genomes.	Viral genome sequences	Annotated genomes with gene functions	Supports functional virology research	Limited coverage for novel genes	Annotation confidence score	^259^
26	VirFinal	Workflow integrating multiple tools for viral genome assembly, annotation, and classification.	Viral NGS data	Complete annotated viral genomes	Integrative, comprehensive	Requires multiple dependencies	Assembly coverage threshold	^260^
27	Angua	Automated tool for viral sequence curation and annotation using alignment and comparative genomics.	Viral genomic sequences	Curated and annotated viral genomes	Reduces manual curation, improves annotation quality	Requires reference genomes for accuracy	Annotation score threshold	^261^
28	Truffle	Alignment-free virus detection tool using machine learning and k-mer based sequence signatures.	Metagenomic reads	Detected viral sequences with confidence scores	Fast, alignment-free, scalable	Limited to known viral families in training	Confidence cutoff ( ≥ 0.5)	^262^
29	Kaiju	Classifies metagenomic sequences based on protein-level alignments using FM-index.	Raw metagenomic reads (FASTA/FASTQ)	Taxonomic classification of reads	High sensitivity for viruses and microbes, protein-level detection	Requires large reference database, memory-intensive	Minimum match length, score threshold	^263^
30	Galaxy	Open, web-based platform for reproducible bioinformatics analysis with workflows for viral genomics.	Sequence data in various formats	Results depending on selected tools (assembly, annotation, etc.)	Highly customizable, reproducible workflows, large community support	Requires server/online setup, can be slower for large datasets	Tool-specific thresholds (e.g., alignment E-values)	^264^
31	ID-Seq	Integrative method for detecting viruses from sequencing reads using statistical modeling and homology searches.	NGS reads	Identified viral sequences and confidence scores	Integrates multiple detection approaches, high accuracy	Computationally heavy, requires good-quality data	Statistical significance cutoffs (*p*-value < 0.05)	^265^
32	Geneious Prime	Commercial bioinformatics software for molecular biology, sequence alignment, and viral genome analysis.	DNA/RNA sequences	Alignments, assemblies, annotations, phylogenies	User-friendly interface, integrates many tools	Paid software, resource-intensive for very large datasets	Software default thresholds (user-adjustable)	^266^
33	CLC Genomics Workbench	Commercial integrated software for genomics, metagenomics, and viral sequence analysis.	Sequence data (raw reads, contigs)	Assemblies, variant calls, annotations, phylogenetic trees	Comprehensive toolkit, supports large datasets, good visualization	Expensive license, requires high system resources	Default quality score and mapping thresholds	^267^

#### Genome organisation and gene content

A primary focus of comparative genomics is examining genome structure and gene content. Viral genomes usually comprise conserved genes whose order and arrangement, known as synteny, reflect evolutionary connections. These features have traditionally been utilized in the informal taxonomic classification of viruses. With the advent of computational methodologies, tools such as VConTACT ^129^and GRAViTy^130^ have been created to evaluate gene-sharing networks and overall genome structure. These tools group viral genomes into related clusters, aiding in formal classification at the family, genus, and even sub-genus levels. Nevertheless, challenges remain for genomes that are highly divergent, where synteny may be disrupted, or situations where there are no universally conserved marker genes, complicating phylogenetic analysis and taxonomic clarification^131^.

#### Phylogenetic approaches

Phylogenetic analysis continues to be a fundamental aspect of comparative genomics, as it assists in reconstructing evolutionary relationships among viruses. Conserved protein domains, such as those encoding capsid proteins, RNA-dependent RNA polymerases (RdRp), or reverse transcriptases, are often utilized as molecular markers for these analyses. Advanced programs like RAxML^132^, IQ-TREE^133^, and BEAST^134^ empower researchers to construct phylogenetic trees based on maximum-likelihood or Bayesian inference algorithms. These trees offer insights into viral ancestry, lineage diversification, and long-term evolutionary patterns. However, challenges persist within phylogenetic methods in virology due to the swift mutation rates of viral genomes, frequent recombination, and horizontal gene transfer phenomena. These dynamics can produce incongruent phylogenies based on the selected gene or genomic region for analysis^135^. To tackle these difficulties, sophisticated tools like VicTree, which operates on multi-rate Poisson Tree Processes, have been created to enhance species-level resolution and mitigate errors stemming from conflicting phylogenetic signals^136^.

#### Pairwise distance-based classification

Pairwise sequence comparison is another prevalent method in comparative genomics. This approach measures genetic distances between viral genomes to determine appropriate thresholds for taxonomic classification. Software such as PASC^137^ and SDT^138^ are designed to rapidly compute sequence identities and produce pairwise similarity matrices, enabling quick and effective classification. Nonetheless, their accuracy is significantly dependent on the composition of the dataset and the depth of sampling. Biased or incomplete reference databases can result in misclassification or an undercount of viral diversity. To address these challenges, more sophisticated frameworks like DEmARC^139^ and VICTOR^140^ have emerged. These methodologies integrate intergenomic distance measures with phylogenetic context, fostering more consistent and hierarchical clustering of viral taxa at the levels of species, genus, and family. Such integrated approaches are especially beneficial when dealing with large monophyletic viral groups.

#### Mapping and reference-based approaches

Mapping techniques are crucial in comparative genomics. In these methods, viral reads or assembled contigs are aligned with existing reference genomes to identify and annotate viral sequences. Commonly utilized tools include Bowtie2^141^, BWA^142^, and Pathoscope^143^. These mapping techniques provide high sensitivity and can detect low-abundance viral sequences within complex metagenomic datasets. However, their effectiveness heavily relies on the availability of suitable reference genomes and the selections made regarding mapping parameters. Importantly, mapping approaches cannot identify truly novel viruses that lack close relatives in reference databases. Therefore, mapping is most productive when used alongside de novo assembly and other discovery-oriented methods.

#### Evolutionary and epidemiological insights

Comparative genomics offers more than just classification and taxonomic organization; it also yields critical insights into viral evolution, epidemiology, and interactions between hosts and viruses. For example, phylogenomic analyses of conserved genes like RdRp in RNA viruses have uncovered the deep evolutionary roots of significant viral clades^144^. Furthermore, comparative genomics has proven to be essential for genomic surveillance of viral outbreaks. During the Ebola epidemic from 2013 to 2016 and the Zika virus outbreak from 2013 to 2014, real-time genome sequencing combined with phylogeographic analyses allowed researchers to reconstruct the dynamics of viral transmission and patterns of geographic dispersal^145,146^. These studies illustrate the rise of phylodynamics, a field that merges comparative genomics with epidemiological data to track viral epidemics in real time.

#### Implications for taxonomy and bioinformatics

The fusion of comparative genomics with advanced computational methods is transforming viral taxonomy and classification methodologies. The ICTV increasingly depends on genome-based approaches for taxonomic determinations, as these methods provide more objective and all-encompassing evaluations of viral diversity^131^. Computational platforms like GRAViTy, VConTACT, and DEmARC have become pivotal to this transition, allowing researchers to define viral taxa with greater accuracy while adapting to the unique evolutionary dynamics of viruses, such as high mutation rates and inconsistent genome sampling. As genome databases grow and bioinformatics methods advance, comparative genomics is anticipated to enhance our comprehension of viral diversity, evolution, and ecological significance further.

### Structural genomics and protein domain searches in viral research

Structural genomics is essential for understanding the organization, function, and evolution of viruses, including plant viruses. By predicting and analyzing protein domains, viral structures, and genomic patterns, this method offers critical insights into viral classification, detection, and the identification of novel viruses. Recent progress in this area combines traditional structural genomics techniques with cutting-edge computational tools, such as ML, to improve sensitivity and precision in viral studies.

#### Protein domain searches, predictions, classifications

A fundamental aspect of structural genomics is the analysis based on protein domains. Unlike standard nucleotide-level similarity searches, protein domain searches concentrate on finding conserved motifs within amino acid sequences, which can often be recognized even within highly divergent viral sequences. Generally, nucleotide sequences are translated across all possible reading frames, and the resulting protein sequences are compared against Hidden Markov Models (HMMs) of known domains using tools like HMMER and HMMScan. Frequently used databases for these searches include the Protein Families database (PFAM)^147^, viral profile HMMs (vFAM)^148^, and the Conserved Domains Database (CDD)^149^. Compared to traditional BLAST-based homology searches, approaches based on HMMs are not just computationally efficient but are also particularly adept at identifying distant homologs^150^ making them crucial for discovering novel or highly divergent viruses. Similar to BLAST, searches based on HMM use statistical significance thresholds (e-values) to assess the reliability of matches^151^. Determining these thresholds requires careful consideration: overly rigid cut-offs may lead to the failure to detect divergent viruses, while more relaxed thresholds increase the likelihood of false positives. Therefore, accurately calibrating e-values is essential for obtaining robust and trustworthy results. One alternative to traditional BLASTx annotation separates the translation phase from classification, specifically utilizing structural and functional characteristics of protein-coding regions. Gene prediction is commonly performed using tools like Prodigal^152^ and TransDecoder, with Prodigal typically demonstrating greater stability across a variety of datasets. The predicted proteins are then classified utilizing tools like usearch or DIAMOND BLASTp against extensive protein databases, followed by HMM-based searches against dedicated repositories such as mcl_4.0^116^.

#### Validation through benchmarking datasets

Benchmarking analyses have shown that protein predictions from both DNA and RNA viruses (excluding viroids) can achieve very high sensitivity, often nearing 100% detection accuracy under most assembly and host removal scenarios. For DNA viruses, DIAMOND generally surpasses usearch, whereas both tools show comparable performance for RNA viruses. However, predictions produced by TransDecoder typically reveal lower sensitivity. Notably, HMM-based searches against the mcl_4.0 database demonstrate strong detection capabilities, despite having fewer sequences than wider repositories like nr_derep1 and nr_derep2. At the species and strain level, some databases showcase distinct advantages; for instance, mcl_4.0 outperforms its source database genome_prot at the species level (median sensitivity ~98.2% compared to ~90.5%), while nr_derep1 and nr_derep2 achieve even greater sensitivity ( ~ 99.3%). Nonetheless, HMM-based methods usually necessitate a supplementary BLASTp-based taxonomic assignment step, as they cannot directly assign taxonomy. This restriction diminishes their practicality for rapid diagnostic uses, but their enhanced ability to detect highly divergent or novel viral sequences highlights their significance in structural genomics. Benchmarking datasets, such as those from the VIROMOCK challenge, thoroughly validate these workflows. These datasets incorporate real, synthetic, and artificially spiked viral sequence libraries, offering a rigorous framework for evaluating bioinformatic pipelines. Researchers’ quality-filter and assemble sequences utilizing tools such as MegaHIT, SPAdes, and virus-specific workflows like rnaSPAdes or rnaviralSPAdes, based on the type of virus. They subsequently annotate the assembled contigs using both nucleic acid–based and protein-based methods, including predictions made by Prodigal. If implemented across diverse datasets and assembly strategies, HMM- and protein domain–based techniques could significantly improve the detection of target viruses.

## ML approaches in plant virology

**Fig. 4:**
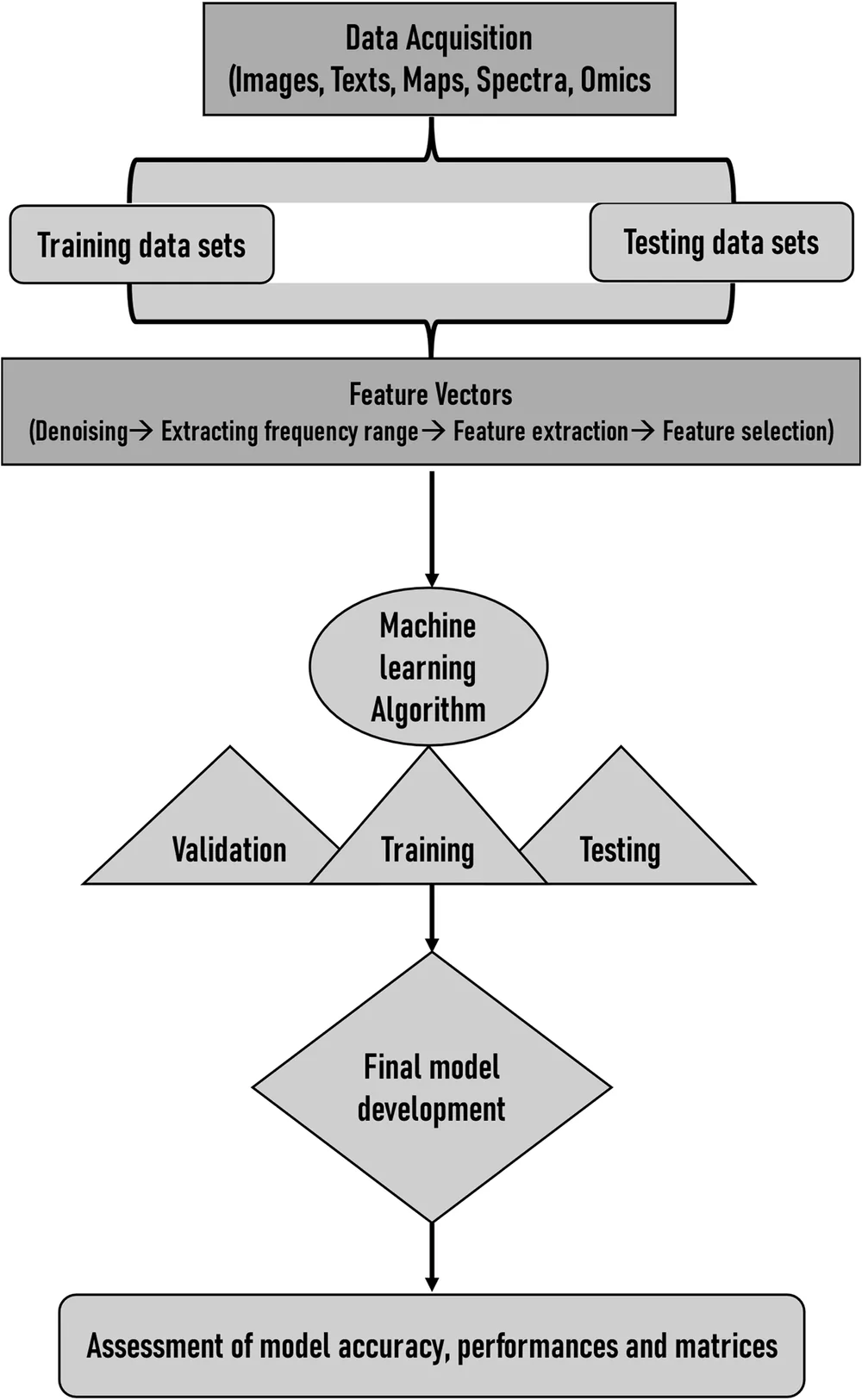
Machine learning workflow in plant virology. The data is acquired from images, maps, texts, etc., split into training and testing sets. Every single piece of data is analyzed on the basis of a feature vector before entering the machine learning algorithm. After that, a resultant machine learning model is generated, which is later evaluated under accuracy, performance and matrix parameters.

The rapid progression of sequencing technologies has led to an exponential increase in biological data, especially in the area of plant virology. Conventional methods of sequence alignment and taxonomic classification, although effective, encounter limitations when applied to large-scale metagenomic datasets. ML, with its capability to recognize patterns within high-dimensional data, presents promising opportunities for detecting, classifying, and predicting viral sequences. Nucleotide k-mer analysis is among the earliest techniques used to annotate sequences by assessing the frequency and distribution of specific k-mers. This method is computationally efficient compared to alignment-based approaches, though it requires considerable memory resources. Tools such as Kraken^153,154^, Kaiju^155^, and Taxonomer^156^ can be adapted for plant virus detection, even though they were not explicitly developed for that purpose. Notably, Kodoja^157^ combines several k-mer tools for the classification of plant viruses in metagenomic datasets. Despite their promise, k-mer methods remain relatively new in the field of plant virus research, raising concerns about their applicability to small RNA datasets and their ease of use. ML models go beyond merely analyzing k-mer frequencies and focus on crafting statistical representations of biological data. Essentially, ML algorithms strive to generalize from pre-existing data, recognizing patterns that enable precise predictions on novel, unseen datasets (Fig. 4). These methods are generally divided into supervised, unsupervised, and semi-supervised learning strategies^20^.

- **Supervised learning** depends on labeled datasets, where algorithms like support vector machines (SVM), random forests (RF), decision trees (DT), and gradient boosting are trained to differentiate between viral and host sequences. Within genomics, features such as genome size, amino acid composition, or metadata (e.g., host species, sampling environment) act as predictors. A typical workflow involves partitioning the dataset into training, validation, and test sets, commonly in an 80:10:10 ratio, to ensure an unbiased evaluation of the model.
- **Unsupervised learning** aims to reveal inherent patterns in unlabeled data. Methods including principal component analysis (PCA), t-distributed stochastic neighbor embedding (t-SNE), and clustering techniques (e.g., k-means, Gaussian mixture models, DBSCAN) are frequently utilized to reduce the dimensionality of data and visualize intricate datasets. These strategies are particularly beneficial for uncovering hidden structures in metagenomic datasets without any prior assumptions.
- **Semi-supervised learning** connects these paradigms by utilizing a small amount of labeled data along with extensive unlabeled datasets. For instance, in the classification of protein sequences, only a minor percentage of sequences might have known annotations, while a greater number remain unclassified. Semi-supervised learning algorithms can take advantage of this disparity to enhance model robustness.

### Applications of DL and ML in plant virology

#### DL and representation learning

Recently, the swift progression of deep learning (DL) has revolutionized the analysis of biological data. DL methods, based on multi-layer artificial neural networks (ANNs), can learn intricate and non-linear relationships without requiring extensive manual feature engineering. This capability makes them particularly effective for processing large-scale omics datasets, often too complex for standard ML techniques. Architectures such as convolutional neural networks (CNNs), recurrent neural networks (RNNs), variational autoencoders (VAEs), and generative adversarial networks (GANs) have emerged as essential tools in both human and plant virology. Frameworks initially created for human virology—like ViraMiner^158^, DeepVirFinder^159^, and VirNet^160^, have shown the ability to accurately distinguish viral sequences from host backgrounds. Although these tools were not tailor-made for plants, they possess significant potential for adaptation in detecting plant viruses (Table 5). However, a significant challenge remains in the form of limited high-quality, curated reference datasets in plant virology, complicating the training of data-hungry DL models. Furthermore, the substantial computational demands of these models continue to pose a practical challenge in environments with limited resources.

**Table 5:** Deep learning models used in plant virology

S.No.	Model name	Working methodology	Mathematical formula	Accuracy (%)	Application in plant virology	References
1	Convolutional Neural Networks (CNNs) – e.g., Inception-v3, ResNet50, VGG19, EfficientNet, DenseNet, Xception, MobileNet	Extract spatial features via convolution, pooling, activation (e.g., ReLU), followed by fully connected layers for classification.	y=softmax(W⋅f(x)+b)y = \text{softmax}(W \cdot f(x) + b)	Up to **99.35%**	Image-based detection of viral and other plant diseases, anomaly detection	^268^
2	Generative Adversarial Networks (GANs) – particularly DCGAN for data augmentation	Generator creates synthetic images, discriminator evaluates realism; helps augment datasets for training.	minαGmaxαDV(D,G)=Ex∼pdata[logαD(x)]+Ez∼pz[logα(1 − D(G(z)))]\min_G \max_D V(D,G) = \mathbb{E}_{x \sim p_\text{data}}[\log D(x)] + \mathbb{E}_{z \sim p_z}[\log(1 - D(G(z)))]	**94.33%** average accuracy when combined with GoogLeNet	Augmenting plant disease datasets (e.g., tomato leaf disease), enhancing classifier performance	^269^
3	YOLO (You Only Look Once) – e.g., YOLOv4, YOLOv5, YOLOv8	Real-time object detection using single-pass CNN: grid cell → bounding boxes + class probabilities.	Output: [x,y,w,h,pclass][x, y, w, h, p_{\text{class}}] per grid cell. Loss combines localization and classification.	96-99%	Real-time detection and localization of viral disease symptoms on leaves, enabling early treatment	^270^
4	Support Vector Machines (SVMs)	Finds optimal separating hyperplane in feature space; uses kernel trick for non-linearity.	minαw,b12∥w∥2 + C∑i = 1nξi\min_{\mathbf{w}, b} \frac{1}{2} \|\mathbf{w}\|^2 + C \sum_{i = 1}^{n} \xi_i, subject to yi(w⋅ϕ(xi)+b)≥1−ξiy_i(\mathbf{w} \cdot \phi(x_i) + b) \ge 1 - \xi_i	**98.79%** (GLCM features, 10-fold CV)	Classification of healthy vs infected leaves based on color/texture features, including viral symptoms	^178^
5	Recurrent Neural Networks (RNNs)	Maintains hidden state to process sequential data and predict future patterns.	ht=σh(Whxt+Uhht−1+bh)h_t = \sigma_h(W_h x_t + U_h h_{t-1} + b_h)	-	Modeling virus spread over time; analyzing gene expression or sequential genomic data	^271^
6	Variational Autoencoders (VAEs)	Encodes data into probabilistic latent space, reconstructs via decoder; useful for generative modeling.	(\mathcal{L} = \mathbb{E}_{q(z x)} [\log p(x z)]- D_{\mathrm{KL}}(q(z x) | p(z)))	-	Modeling virus spread over time; analyzing gene expression or sequential genomic data	
7	Decision Tree	Tree-based classification using feature splits; interpretable.	Impurity minimization: minαj,θ ∑cwc Impurity(Sc)\min_{j,θ} \; \sum_{c} w_c \, \mathrm{Impurity}(S_c)	~74–75%	Used in plant virus suppressor classification.	^272^
8	XGBoost	Gradient boosting of trees with regularization.	y^i = ∑kfk(xi), L = ∑il(yi,y^i)+∑kΩ(fk)\hat y_i = \sum_k f_k(x_i), \; \mathcal{L} = \sum_{i} l(y_i,\hat y_i)+\sum_k \Omega(f_k)	Upto 90%	Virus strain detection, Raman spectral classification.	^273,274^
9	ElasticNet / Lasso / Ridge	Linear models with L1/L2 penalties.	minαw 1n∑(yi−wTxi)2 + λ1∥w∥1 + λ2∥w∥22\min_w \; \tfrac{1}{n} \sum (y_i - w^Tx_i)^2 + \lambda_1 \|w\|_1 + \lambda_2 \|w\|_2^2	-	Biomarker selection from plant virus transcriptomics.	
10	Random Forest	Ensemble of decision trees (bagging + feature randomness).	y^=mode{T1(x),…,TM(x)}\hat y = \mathrm{mode}\{T_1(x),…,T_M(x)\}	~86-97%	Early virus detection, biomarker importance analysis.	^275^
11	MLP	Neural network with hidden layers and backpropagation.	h(l)=σ(W(l)h(l − 1)+b(l))h^{(l)}=\sigma(W^{(l)}h^{(l-1)}+b^{(l)})	~85–90%	Virus-related classification from gene expression.	
12	SGD	Optimization algorithm for training ML models.	w ← w−η∇wℓ(w;xi,yi)w \leftarrow w - \eta \nabla_w \ell(w;x_i,y_i)	-	Used in training CNN/LSTM for virus classification.	
13	LSTM	RNN variant with memory gates for sequence data.	Gates: it,ft,ot,ct,hti_t, f_t, o_t, c_t, h_t equations.	~93.4%	Time-series infection data, hyperspectral sequences.	^276,277^
14	Reinforcement Learning	Learns policies via reward feedback.	J(π)=E[∑t=0Tγtrt]J(\pi)=\mathbb{E}[\sum_{t = 0}^T \gamma^t r_t]	-	Potential: optimize virus sampling/control strategies.	
15	BERT	Transformer-based contextual LM; bidirectional self-attention.	Attention(Q,K,V)=softmax(QKT/dk)V\text{Attention}(Q,K,V)=\mathrm{softmax}(QK^T/\sqrt{d_k})V	~85-95%	Symptom text analysis, multimodal virus detection.	^278^,^279^

#### Model evaluation and challenges

The effectiveness and dependability of ML and DL techniques are heavily reliant on comprehensive evaluation metrics. Frequently referred indicators include accuracy, precision, recall (sensitivity), specificity, F1-score, and confusion matrices. Despite their promising capabilities, DL models can suffer from overfitting (capturing irrelevant noise instead of genuine patterns) or underfitting (failing to capture sufficient complexity). To mitigate these issues, researchers utilize approaches such as dropout regularization, batch normalization, early stopping, and cross-validation. Additionally, plant virology datasets introduce further challenges. They are frequently small, skewed towards a limited number of model host–virus systems, and characterized by high dimensionality. Consequently, performance may seem exaggerated in benchmarking tests but may not generalize effectively across less-studied viruses. Addressing such challenges necessitates the creation of balanced and representative datasets, along with integrative approaches that combine diverse data types (e.g., genomic, transcriptomic, and imaging data)^161,162^

#### Applications in detecting plant diseases

Conventional methods for diagnosing viral diseases in plants primarily depend on expert assessment of visible symptoms. While these approaches can be effective in specific situations, they are often time-consuming, require significant labor, and may lead to misinterpretations influenced by environmental conditions. Classical ML techniques—such as support vector machines (SVM), decision trees (DT), random forests (RF), and k-nearest neighbors (KNN)—have been utilized to automate this diagnostics process through the use of image-based features^163–165^. Despite offering useful insights, these techniques’ heavy reliance on manually crafted feature extraction restricts their scalability and accuracy across varying field conditions. DL, particularly CNN-based frameworks, has overcome many of these challenges by automatically learning distinguishing features from raw images. Architectures like AlexNet, VGGNet, ResNet, EfficientNet, and DenseNet have shown impressive results in identifying plant leaf diseases across different environmental circumstances^166–168^; (Recent developments even incorporate DL-enabled object detection into drone systems for large-scale surveillance, presenting the potential for real-time monitoring of crop health. However, issues such as varying lighting, leaf occlusion, and complex backgrounds still pose significant challenges.

The strength of DL lies in its superior ability to handle unstructured and multidimensional data compared to traditional ML techniques. Deep networks progressively extract features at several levels—from basic pixel arrangements to complex abstract representations—via layered perceptron structures^169,170^. This multi-level representation minimizes the chance of models becoming stuck in local minima while eliminating the need for manually designed features. As a result, DL has been widely applied not only in plant virology but also in areas such as recommendation systems, computer vision, speech recognition, and natural language processing^171^. One of DL’s primary benefits is its resilience in image recognition tasks. In contrast to traditional image classification techniques that depend on manual feature extraction, DL methods utilize back propagation to iteratively refine features, allowing them to capture local details and contextual information^172,173^. This feature is especially important for recognizing intricate symptoms of plant diseases. New architectures, including stacked denoising autoencoders (SDAEs), deep belief networks (DBNs), deep Boltzmann machines (DBMs), and advanced CNNs, further broaden the potential for precise and automated disease detection^174^.

### ML for detecting plant diseases through image processing

The process of diagnosing and classifying plant diseases using ML combined with image processing typically consists of a five-stage pipeline: image acquisition, image preprocessing, image segmentation, feature extraction and selection, and classification^175^. Each stage is vital for ensuring that infected plant tissues are identified accurately and reliably.

#### Image acquisition

The initial phase in any ML-driven disease detection system is the acquisition of images. Images capturing plant components such as roots, stems, branches, and leaves can either be taken directly or obtained from image repositories. The quality of the acquired images significantly affects the performance of the system and ultimately influences the accuracy of disease detection^176^. Factors such as shadows, background noise, and poor lighting can compromise the reliability of the captured images. To address these issues, pre-processing techniques like noise and background removal are utilized before further analysis. While conventional RGB cameras are commonly employed, specialized imaging methods—like hyperspectral, thermal, and fluorescence imaging—provide considerable benefits by uncovering subtle disease indicators not detectable in standard images. Nevertheless, images captured in the field tend to show greater variability than those taken in controlled laboratory conditions, as environmental factors (e.g., erratic lighting and complex backgrounds) introduce distortions. Thus, the techniques and equipment used during image acquisition have a significant impact on the effectiveness of ML-based detection systems.

#### Image pre-processing

Pre-processing serves as a vital stage that improves image quality by correcting distortions, interference, shadows, and other degradations^177^. Since datasets are usually collected in uncontrolled, real-time environments, pre-processing boosts the accuracy of the subsequent feature extraction and lessens computational load. Standard pre-processing tasks include cropping, resizing, background removal, and image enhancement. Furthermore, image augmentation techniques are often applied to artificially increase the size of the dataset, which is particularly beneficial for DL models that require substantial amounts of training data. Augmentation strategies comprise noise injection, flipping, gamma correction, scaling, rotation, random shifting, zooming, contrast adjustment, and brightness changes. These procedures enhance model robustness and mitigate the likelihood of overfitting.

#### Image segmentation

Image segmentation focuses on isolating the region of interest (ROI)—specifically, the diseased area of the plant—from the healthy background. By defining the infected section, segmentation facilitates further analysis and enhances classification accuracy. However, factors such as shadow interference, varying illumination, and complex backgrounds pose challenges to this procedure. Segmentation methods can be broadly categorized into two types^178^.

- **Conventional techniques**, including thresholding, region growing, and edge detection.
- **Computational methods**, such as fuzzy logic, genetic algorithms, and neural networks.

While traditional methods are straightforward and cost-effective in terms of computation, advanced computational techniques typically provide better accuracy in differentiating healthy from diseased regions. The chosen segmentation approach has a direct effect on the quality of features extracted and, ultimately, the effectiveness of disease classification.

#### Feature extraction and selection

Feature extraction is a crucial component of computer vision and ML systems, enabling the differentiation between healthy and diseased tissues by quantifying image attributes. Commonly extracted characteristics include color, shape, and texture, which deliver essential information for identifying diseases. For example, spotted areas are analyzed primarily using texture and color features, while shape descriptors help capture morphological alterations in leaves. Given the overlap of symptoms among various diseases, it is vital to select the most pertinent features to minimize redundancy, cut down computational costs, and prevent overfitting. Feature selection methods like PCA, genetic algorithms, and particle swarm optimization are often utilized to refine the feature set. Recent literature highlights the growing diversity of feature extraction methods applied to different crops over the last decade, demonstrating the adaptability of ML techniques to varying agricultural contexts. Nevertheless, feature determination remains a major research challenge due to overlapping symptom characteristics across diseases.

#### Classification

The concluding phase of the ML pipeline is classification, where the system assigns a label (e.g., healthy or diseased) to the input image based on the features extracted. The success of classification is significantly influenced by the earlier steps of acquisition, preprocessing, segmentation, and feature selection. Typically, a dataset is split into training and testing subsets, allowing the system to recognise disease-specific patterns before being tested on new images. ML, a subset of AI, allows systems to autonomously learn and enhance performance from data without direct programming^179^ Classification methods can be generally divided into three categories: supervised learning, unsupervised learning, and semi-supervised learning. Frequently utilised classification algorithms for detecting plant diseases include support vector machines (SVMs), ANNs, and k-NN. Moreover, fuzzy logic and vegetation index-based methods have been investigated for particular applications. In the realm of ANN-based models, variations such as feed-forward neural networks, multilayer perceptrons, probabilistic neural networks, error backpropagation networks, and self-organizing maps have been widely utilized. These models exhibit a high level of adaptability to the intricate and varied symptomology seen in plant diseases.

## Exploring the challenges and considerations of utilizing plant viruses in biotechnology applications

Plant viruses have been utilized as biotechnology tools and are now being expanded for improvement in agriculture and industrial biotechnology, for use as vaccines, as gene delivery systems, nanotechnology applications, and several other exciting avenues of research. Nevertheless, this rapid progression presents complex dilemmas associated with biosafety, biosecurity, and regulatory compliance. We must be concerned about these issues because of the danger plant viruses may pose when they spread and persist in cultivated and wild plants^8,10^. This section discusses two main elements, biosafety and biosecurity (6.1) and regulatory frameworks (6.2) that guide the responsible use of plant viruses in various biotechnological applications.

### Biosafety and biosecurity

Biosafety involves the containment principles, technologies, and practices implemented to prevent unintentional exposure to pathogens or their accidental release, while biosecurity focuses on preventing the misuse of these biological agents, including intentional release or bioterrorism.

#### Unintended spread and environmental concerns

Plant viruses, including RNA viruses such as TMV, PVX, CPMV, etc., are usually used as tools for gene expression, VIGS, or the construction of nanocarriers. They move systemically and efficiently and can duplicate and spread in plants, making them unprecedented bio tools. However, this movement can frequently lead to inadvertent spread to non-target species via horizontal transmission, especially in an open-field application^180^. For example, if a recombinant PVX vector was used in a greenhouse/trial, the vector can potentially infect unsuspecting hosts with the aphid vector or through mechanical contact and situate the engineered construct in another uncontrolled environment. Also, the RNA viruses are quite plastic and may recombine with a wild-type virus and yield other pathovars, perhaps generating another pathogenic strain^181^.

#### Containment measures and host range

The establishment of robust containment measures when utilizing plant viruses in biotechnology is vital because they can naturally replicate, spread systemically, and can be transmitted to a non-target host. Containment measures designed to prevent the release of a virus are important in protecting not just cultivated crops but also preventing an unintentional environmental disturbance by a virus that may find its way out into the wild^17^. An important part of containment is to try to nefariously convey virus-mediated traits without propagating the virus itself, and this requires good design of the vector. This could be accomplished around the construction of a virus with inactivated movement proteins, CPs, or whatever genes are essential for its propagation or movement. The viral vector could be rendered incapable of replicating or translocating propagated unless complemented in trans by a helper construct^182^. For instance, mutations have been incorporated into many recent vectors that utilize Tobamoviruses or Potexviruses to stop movement by cell-to-cell or long-distance transport, which defines the area of dissemination within the host plant and potentially adjacent plants^183^. A primary method includes physical restriction to controlled areas such as laboratories or greenhouses, and designated biosafety levels (typically BSL-1 or BSL-2 for most plant viruses)^184,185^. Laboratories have airlocks, double doors, and personnel with decontamination processes to minimize accidental release. Greenhouses working with recombinant viruses may also include insect-proof barriers, soil sterilization, and waste disposal methods to reduce the potential for transmission by vectors. Many researchers elect to work with model plant species that do not have known relatives in the local area, which also reduces the potential for infection between species^186^.

The wide range of hosts entails significant implications for containment strategies. Plant viruses that have wide host ranges pose a greater risk for containment, in addition to more strict provisioning management than those that infect a few species (Table 6). For this reason, it is prudent to either select or engineer viruses that attain narrow host specificity as an effective biosafety strategy^187^. A wider and more varied host range has significant implications for control strategies. Plant viruses that infect a greater diversity of hosts will be more difficult to manage and require greater control measures than viruses that infect only a few hosts. Therefore, it is prudent to select or create viruses with a narrow host range as a measure of biosafety. This would reduce the risk of undesirably infecting non-target or wild species, and we would expect a lower ecological impact from our applications of viral vectors. For example, it has been argued that, ideally, viral vectors should be limited to the host range to guide environmental dispersal and genetic interaction with wild-type viruses. Furthermore, viruses that are limited to fewer hosts will reduce the likelihood of wild viruses recombining with viral vectors to make new, unpredictable, more virulent, and more diverse strains with expanded host ranges^188^. Another way to provide additional biosafety is to utilize genetically engineered plant lines, which restrict viral replication (e.g. by way of RNA silencing of pivotal viral genes). Furthermore, important information exists in a correlation of viral biology and environmental factors (e.g. temperature, humidity and insect vectors) when formulating containment strategie^189,190^. In summary, containment can also consist of not just physical barriers but also biological barriers using the virus in question. A combination of physical barriers and biological barriers makes removal of the action of escape difficult. Even if escapes occur, then the chance of establishing viral escape in the ecosystem is likely to be low.

**Table 6:** Widely used plant viruses in biotechnology, concerning their host range, applications, and necessary containment strategies

Virus	Host range	Application	Containment required
Tobacco Mosaic Virus (TMV)	Broad	Gene delivery, nanotechnology	BSL-2, insect-proof greenhouse
Potato virus X (PVX)	Moderate	VIGS, protein expression	BSL-1/2, limited host range
Cowpea mosaic virus (CPMV)	Narrow	Nanocarriers, vaccines	BSL-1, soil decontamination

#### Risk assessment and monitoring

Assessing risk is essential to managing biosafety when utilising plant virus-based technologies in biotechnology. Risk assessment is an evaluation of the likelihood and consequences of accidental exposure, release into the environment, and long-term ecological impacts. A critical component of risk assessment is to characterise the viral vector. Important characteristics to assess include genomic stability, breadth of host, means of transmission, and the possibility of recombining with endemic viruses^191^. For example, vectors based on single-stranded RNA viruses like PVX or TMV have quite high mutation rates. Because of this, there are valid concerns over the emergence of novel variants with unpredictable properties. Second, viral vectors such as PVX and TMV can infect with systemic symptoms, and be transmitted by vectors, allowing accidental release of the vector in species beyond the intended plant species^12^. Another key knowledge item in the entire risk assessment process is the assessment of engineered traits. Once transgenic viruses have been engineered to express a foreign gene, be it a reporter protein, a peptide for pharmaceutical purposes, or an insecticidal factor, the biological property of the transgene has to be assessed for potential toxicity, allergenic potential, or ecological consequences^192^. An example of this is a virus engineered to express an insecticidal protein that is consequently released into the environment through infected plant tissue and contaminates beneficial insects. For this reason, molecular characterization of each construct during risk assessment, including aspects such as transgene copy number, gene expression, and gene insertion stability, has to be assessed before field use^193^.

Post-assessment monitoring is crucial to continuing biosafety beyond deployment. Environmental monitoring programs should be put in place to assess the potential for viral spread of the product beyond the targeted area, including the use of molecular diagnostics (RT-PCR and qPCR) to measure viral RNA in non-target plant species and potential vector species^194^. Regular assessments of plant populations within the buffer zone adjacent to, and especially surrounding the test site, can forward momentum in the status of potential off-target infection. The monitoring context also includes the assessment of viral RNA, or CPs, in soil, water, or decomposing plant debris, which can also affect later planting cycles or non-target organisms. Additionally, ongoing monitoring provides some level of accommodation for unanticipated development (e.g., climate change, mutation events, introduction of new host species). In addition to preparing field evidence from detailed pre-release risk assessments and concurrent monitoring of the released species (and surrounding environment), this approach may be advantageous for risk mitigation and allows for both regulators and researchers to use real-time data to act quickly for the possible mitigation of risks in the environment^195^. In the evolving area of plant virus biotechnology, this type of environmental oversight is vital and responsible innovation.

### Regulatory frameworks

At present, there are various systems of governance in place to control the use and development of biotechnologies exploiting plant viruses (i.e., plant virus-based biotechnologies). These systems were developed to manage risks to human/animal health and the environment, and to promote innovations in new technologies. These systems approach the risks associated with genetically modified organisms (GMOs), including those developed with viral vectors, and guide what oversight is required before field release or commercialization. This is especially important for plant viruses; since they can replicate, can spread to a range of host organisms and can also horizontally transfer their genetic material. Regulatory systems are generally clear about the distinction between contained use, e.g., laboratory or greenhouse situations, and intentional environmental release, and hence will require increased oversight and assessment^196^. In practice, plant viruses can create difficulties for the current governance systems because they do not always fit the description of a GMO, and often, in these situations, there is no heritable genetic alteration in the host. This can generate regulatory uncertainty, which can delay or halt the research and development of products. Moreover, plant virus applications also are extremely heterogeneous, ranging from transient expression systems to VLPs that can be used either as vaccines or nanocarriers; thus, regulation should ideally take place on a case-by-case basis, rather than as they do now, as a “one-size-fits-all” policy^197^. As many developers are aware, regulations may be ambiguous, and costs are climbing with long wait times. Upon implementation, disruptive technologies such as CRISPR-based viral vectors, synthetic viroids, and modular expression systems may lack full coverage in existing regulations, requiring context-sensitive and adaptable policymaking. A proper regulatory framework must be weighted to encourage innovation while ensuring biosafety and environmental protection, while at the same time ensuring new technologies are both effective and safe. The policy should be a facilitator of innovation rather than barriers, given that we are facing global challenges such as climate change and food insecurity and should enable and provide meaningful access to new technologies in a balanced, transparent and responsible manner, including new technologies developed incorporating plant viruses^198,199^.

#### Countries-specific regulations

Regulations related to plant virus biotechnology differ greatly among and sometimes even within countries. The disparity in regulations between countries can be a combination of different biosafety regulations, public perceptions about genetically modified organisms (GMOs), the capacity of the institution, and legal frameworks^200^. In the United States, USDA’s Animal and Plant Health Inspection Service (APHIS) is primarily responsible for plant virus vectors, as long as the viral construct comes from a regulated plant pest. The EPA would be involved if the engineered virus had pesticidal properties and the FDA would regulate virus-based products for pharmaceutical or food purposes^201^. In contrast, the European Union (EU) has much stricter regulations under Directive 2001/18/EC, which is the directive for the deliberate release of GMOs including plant virus vectors. EU regulations include an in-depth required environmental risk assessment, public consultation and traceability, even for systems that only express transgenes transiently. The EU’s precautionary turn on GMO issues has hampered field trials and commercialization of viral vectors (especially those expressing transgenes)^202^. In India, the management of plant virus-based technology is regulated under the 1989 Rules of the Environment (Protection) Act of 1986, and this is done by the Genetic Engineering Appraisal Committee (GEAC) within the Ministry of Environment, Forest and Climate Change (MoEF&CC). The Review Committee on Genetic Manipulation (RCGM) under the Department of Biotechnology (DBT) is responsible for the approval of research. Anything involving ‘recombinant’ plant viruses, whether in the laboratory or during field trials, requires biosafety clearance from the GEAC and RCGM to be completed. When the GEAC reviews requests for biosafety approvals for field trials, it assesses and considers the risks relating to viral replication, host range, gene flow, etc. India has a formal regulatory system regulating transgenic organisms; however, criticism is that the processes involved can be slow and mired in bureaucracy, which might inhibit innovative and practical uses of technology^203^. Some countries, including Brazil, China, and Australia, apply different regulations on GMOs to their plant virus vectors, but the clarity and implementation of those regulations may differ. Current inconsistencies with countries when undertaking regulatory definitions of ‘non-integrative’ or transient systems highlight the case for aligned, evidence-based policy reform.

#### Challenges in regulatory oversight

The regulation of biotechnologies utilizing plant viruses faces a variety of serious challenges, in part due to the rapid pace of technology itself. One of the challenges is poor regulatory definitions; for example, should transient expression systems using plant viruses be treated as GMOs, as there is no stable incorporation into the genome? Such poor definitions often lead to variable regulatory decisions, or worse, risk avoidance strategies that do not accurately reflect the actual risks^204,205^. Regulatory lag is also persistent; innovations such as CRISPR-based viral vectors or synthetic VLPs emerge faster than regulatory bodies can adapt, leading to periods of no regulation^206^.

#### Future regulation and framework

With the introduction of plant virus-based technologies into agriculture, therapeutics, and nanotechnology, there is consensus about the necessity of having a common and flexible global regulatory framework. A common framework that enables international trade and transfer of technology, as well as equitable access to biotechnologies by countries, will also benefit all users. To achieve this, international organizations such as the OECD, WHO, FAO and CBD could work together to develop fundamental safety standards and risk assessment approaches for virus-derived biotechnologies. Development of these standards should be science-based and, where possible, rely on product characteristics rather than the means of production^207^. For example, a product assessment would consider the ecological and human health consequences of a virus-expressed protein, regardless of whether it was introduced into the environment via a virus or a transgene. Furthermore, a flexible framework would facilitate the inclusion of new technologies (for example, self-amplifying RNA systems, synthetic viroids, and programmable virus-based nanodevices), which will not fit easily into existing regulatory categories or governance as technology progresses, thus requiring regulatory bodies to continually evaluate, and determine updated policies and assessments of risk for new technologies^38^. Regulatory frameworks should anticipate and incorporate tiered risk assessment value considering factors such as environmental exposure, host range, viral stability, route of introduction and environmental intent of use. In addition, for permit authorities and other stakeholders to build trust and address ethical/societal issues often raised by the application of virus-based technologies, there must be public engagement and transparency. The establishment of regional centres of regulatory excellence and training regulators in developing countries will also expand the possibilities for oversight. Ultimately, the framework will be a mechanism that does not inhibit innovation but rather provides a pathway to safety, science, and societal value collectively. By promoting collaboration and consistency, the global community can enhance and promote the responsible and effective use of plant virus technologies.

## Future perspectives and directions

The convergence of plant virology with AI, ML, bioinformatics, and nanotechnology presents a fertile ground for unprecedented innovation. While the current applications are promising, the next frontier in this field will be defined by a more integrated and predictive approach, moving beyond empirical discovery toward rational design.

### Emerging technologies

One of the most important future directions will undoubtedly be the viral engineering sophisticated computational platform for viral engineering. Just think about ML algorithms trained on vast datasets of viral capsid structures as well as their assembly kinetics to predict the best mutations for increasing stability, cargo loading efficiency, and targeting specificity^208^. For example, the use of generative adversarial networks (GANs) or deep reinforcement learning can allow us to create new VLPs with tunable properties that suit certain biomedical or agricultural applications^209^. This would be an enormous leap beyond the traditional trial-and-error approach. The researchers will be able to predict the viral capsids' atomic-level operation through the integration of molecular dynamics simulations under different environments. In this way, design alterations can be virtually tested before actual synthesis is made, hence, saving a lot of resources^210,211^. Besides that, the use of single-cell analysis and high-throughput screening will greatly affect the field. The use of microfluidics may be combined with the fluorescent tagging of PVNPs in order to have real-time delivery and expression tracking at the cellular level. This will open the door to training the models using the data collected, which will offer a more significant understanding of virus-host interactions and will help in the therapeutic vector designs. To accomplish, the multi-omics integration will efficiently aid the processing of data obtained through different biological techniques. The ML models are capable of doing such an analysis on genomic, transcriptomic, and proteomic data concurrently and thus find out intricate gene regulation networks and off-target effects previously unknown. The future of the field could also witness the increased use of bio-orthogonal chemistry. The viral capsids could be designed with reactive groups so that the researchers can add a variety of functional molecules, such as drugs, imaging agents, or polymers, to the particle with high accuracy and minimal interference with the structure of the particle^212^. This will make the creation of multifunctional nanodevices from PVNPs easier and faster. Moreover, combining viral nanocarriers with CRISPR-based technologies is another extremely exciting direction, as it allows for specific and highly targeted gene editing in plants for traits such as disease resistance or enhanced nutrient content ^213^Synthetic biology, lastly, is the change-agent that will most likely impact the process. Scientists will no longer be limited to the usage of naturally-occurring plant viruses that have been modified; they could simply come up with entirely new viral systems, whose functions and safety characteristics have been previously defined, thus, from the very inception itself. In fact, the example could be the creation of synthetic viral genomes that do not infect the host, but still can self-assemble into impenetrable nanostructures.

### Potential research areas

Several research areas hold tremendous potential, and the creation of “smart” viral vectors would be a primary focus that could be triggered by a specific environment or physiological condition. To illustrate, a viral nanocarrier may only require a pathogen-derived metabolite to be detected or, alternatively, the tumor microenvironment releases the genetic material in question in order to carry out the therapy. As such, very targeted and instant therapies would be possible. The investigation may go into the development of the light-activated or pH-responsive viral nanocarriers, which will let go of the payload when only exposed to certain wavelengths of light or in the acidic environment of a tumor. Accordingly, the drug delivery of this kind will be completely revolved. Furthermore, another area is the implementation of AI and bioinformatics to delve into the virome for new therapeutic candidates. The uncharacterized diversity of plant viruses is a giant resource that is barely being tapped. One could use ML to sift through metagenomic data to fish out novel viral proteins that have never before been seen and would be used as a source of biotech due to their characteristic of either regulating the immune system or self-assembling, etc. This is especially important when discovering new enzymes or protein domains that only have one catalytic activity that can be used for industrial or medical processes. Last but not least, every effort aimed at resolving biosafety and regulatory problems pointed out in the previous section will take center stage. Creating safe PVNPs with features such as programmed degradation and limited host range will have to be the main focus of research in the next phase. Therefore, risk assessments in which computational models and simulations are employed to also foresee potential off-target effects and environmental influences before the commencement of the field trials could be a method totally suitable for this activity. The establishment of predictive models for viral evolution might be the defining moment as it will enable researchers to simulate how the genetically modified viruses can undergo mutations and interact with other species that are not the target ones, therefore, choosing the best containment strategies to proceed safely with ongoing. Establishing international data-sharing protocols and standardized reporting metrics will also be crucial for building a global framework for the safe and ethical use of plant virus-based technologies.

## Conclusion

Once considered only a major threat to agriculture, plant viruses have become the most highly adaptable and efficient tools for biotechnology. This article has acknowledged the extraordinary utilization of plant viruses as gene conveyers, carriers of vaccines and nanocarriers for varied use spanning from the human health sector to environmental cleanup. Nature has endowed these smallest microorganisms with the necessary features for self-joining, stability, and compatibility with life, thus making them an interesting alternative to artificial nanomaterials. One of the most important steps in the direction of this field's full growth is the joint use of AI, ML, and bioinformatics. There is a considerable transformation of the attitude toward computational disciplines, and they’re actually becoming the core process of new knowledge and design, besides being just helper tools for taking care of big amounts of data. While bioinformatics unveils the details of viral genetics and structure, ML and AI provide the facilitation of viral systems prediction, optimization, and rational engineering. The blending of these disciplines assures the coming of a new era of highly accurate biotechnology where smart algorithms and predictive models lead the creation of virus-based therapy, diagnosis, and agriculture. Such a trend change will find great application with the solutions of the world’s most difficult problems, among which can be mentioned the improvement of global food security through the engineering of crop resistance, as well as the quickest-setting vaccine platforms for fighting newly arisen pandemics. The use of these means will draw the way to more sustainable and efficient solutions, which will allow us to decrease the utilization of traditional chemical pesticides and other resource-demanding methods. Still, there are concerns regarding the safety of these technologies and how they will be regulated that need to be dealt with while carrying out the implementation of the technological frontier. Risk management and honest communication with all parties involved, as well as the establishment of strong and internationally coordinated regulatory frameworks, should be elements that inevitably characterize the incorporation of advanced technologies for biosafety purposes. This will play the most crucial role in gaining the confidence of the public and preventing any unforeseen damage to the ecosystem. The future course of this domain will depend not only on the successful confluence of the enigmatic scientific world but also on the cooperative participation of the virologists, computer scientists, policymakers, and the public Ultimately, by harnessing the elegance and power of nature’s smallest engineers, guided by the immense potential of AI and data-driven insights, we stand on the cusp of a revolution in both human and planetary health.

## Data Availability

No datasets were generated or analysed during the current study.
